# ASGOP: An aggregated similarity-based greedy-oriented approach for relational DDBSs design

**DOI:** 10.1016/j.heliyon.2020.e03172

**Published:** 2020-01-09

**Authors:** Ali A. Amer, Marghny H. Mohamed, Khaled Al_Asri

**Affiliations:** a Taiz University, Yemen; b Assiut University, Egypt; c Hajjah University, Yemen

**Keywords:** Information science, Computer science, Vertical fragmentation, Clustering, Data allocation, Data replication, DDBS, Greedy algorithms, ASGOP

## Abstract

In the literature of distributed database system (DDBS), several methods sought to meet the satisfactory reduction on transmission cost (TC) and were seen substantially effective. Data Fragmentation, site clustering, and data distribution have been considered the major leading TC-mitigating influencers. Sites clustering, on one hand, aims at grouping sites appropriately according to certain similarity metrics. On the other hand, data distribution seeks to allocate the fragmented data into clusters/sites properly. The combination of these methods, however, has been shown fruitful concerning TC reduction along with network overheads. In this work, hence, a heuristic clustering-based approach for vertical fragmentation and data allocation is meticulously designed. The focus is directed on proposing an influential solution for improving relational DDBS throughputs across an aggregated similarity-based fragmentation procedure, an effective site clustering and a greedy algorithm-driven data allocation model. Moreover, the data replication is also considered so TC is further minimized. Through the delineated-below evaluation, the findings of experimental implementation have been observed to be promising.

## 1 Introduction

Over the past forty years, numerous approaches have been evolved in DDBS literature to functionally manage the ever-growing data. Nevertheless, several issues for improving DDBS design quality are still open-ended challenges. Particularly speaking, the relational distributed database which is still growing in popularity. Consequently, the continuous interest is also still emphasized towards finding the well-designed approaches to keep the sustainability of DDBS performance. On the other hand, the most critical contributor in performance is that how much amount of data is being transmitted over the network when distributed queries are under processing. This dominant contributor has widely known as Transmission Costs (TC) for which most of the previous DDBS works had come to find a solution of influential impact. Moreover, it has been noted that almost the majority of earlier works have never been recorded to come up with a clear definition for this contributor by which performance is set to be graded (Amer et al., 2018a, b).

In the meantime, the wealthy existence of design approaches leads to more confusion when it comes to select certain approach to design DDBS. Fortunately, to lessen this burden, a consensus on the principles and concepts which ground these approaches still exist (Amer et al., 2018a, b). Additionally, the ever-progressing researches in DDBS and distributed computing domains are still in the development now and then to tackle the DDBS design challenges. Some of these approaches are subtly optimized or well extended to incorporate other techniques straightforwardly to sustain DDBS throughput (Raouf et al., 2018; Luong et al., 2018; Wiese et al., 2016; Sewisy et al., 2017; Amer, 2018; Abdalla and Artoli, 2019). The findings of these approaches have been reinforced by placing them directly under the test on either synthesized data in a simulated environment or on real datasets. In this work, therefore, we seek to present a new approach with the major purpose of significantly decreasing TC. The proposed approach was essentially designed to involve and develop: (1) an aggregated similarity between queries to fragment data using the single-linkage Agglomerative Hierarchical Clustering (AHC) process, (2) a greedy-based data allocation model to allocate the resulted fragments, and (3) integrate the site clustering procedure which was drawn in (Amer et al., 2017). It is worth indicating that the choice of the single-linkage has been on purpose as our work seeks to join each cluster pair based on the maximum similarity value between the cluster pair. In other words, the closest distance is adopted to define the similarity between each cluster pair. Data allocation, on the other hand, was made using a greedy-based algorithm which is highly contingent on the dynamic programming (Knapsack problem, in this work). That is, data allocation was being treated as an optimization problem. Moreover, a theoretical comparison and empirical evaluation for the proposed work of this paper is made with state-of-art approaches. In fact, the evaluation results have been promising in terms of the sustainability of DDBS performance and TC reduction.

The main contributions of this paper are listed as follows: (1) leveraging data fragmentation algorithm based on an aggregated similarity. The similarity measure is anticipated to reduce the number of iterations needed to perform AHC and then find solution space of smaller size comparing with the state-of-art. Moreover, the proposed approach does not need the query frequency matrix to perform fragmentation, nor affinity matrix or even attribute usage matrix which makes our work the best option to design DDBS at the initial stage of design; (2) drawing the site clustering process to produce minimum number of highly balanced clusters. The clustering process of queries, on the other extreme, struggles to find the balanced cluster as each cluster must have at least (√N) query to help distributing the workload over network sites/clusters while distributed queries are being handled; (3) suggesting a greedy-based data allocation algorithm to contribute in minimizing TC. That is, dealing with data allocation as an optimization problem and finding a model to address this problem. Finally, along this paper, for the sake of readability and simplicity, the proposed approach is given an acronym named “ASGOP”. This acronym, on the other hand, is found by taking the first letters of each word of paper's title as follows; An Aggregated “A”; Similarity-based “S”; Greedy-Oriented “GO” and Approach “P”.

The remaining of this paper is structured as follows; in section (2), a deeply-made investigation for the earlier works which are closely related is presented. Section (3) holds the proposed methodology including data fragmentation procedure, the proposed aggregated similarity measure, AHC review in a brief, fragmentation evaluator, site clustering process and data allocation cost model foundations and algorithms. Section (4) elegantly provides experimental setup of ASGOP, datasets descriptions, the running example to draw the proposed work mechanism and performance evaluation. An experimental implementation along with the discussion of findings are drawn in section (5). Finally, in section (6), the conclusions and future work directions are given.

## 2 Related work

### 2.1 Data fragmentation

Data fragmentation (Vertical, Horizontal or Mixed) has long seen to play the key role in DDBS performance enhancement. As a matter of fact, it is commonly agreed upon that the proper the data fragmentation and allocation (including replication) are, the highly likely that the overall performance of DDBS is sustainably satisfied (Nashat and Amer, 2018). In (Nashat and Amer, 2018), a fine-grained taxonomy was drawn. This taxonomy was extensively examined that more than one hundred references (Chapters, Papers, Reports, Books, etc) were investigated in both static and dynamic environments. The key drive behind this taxonomy was to find the drawbacks and shortcomings from which most of earlier works were observed to be suffering. Data fragmentation, data allocation and replication were all studied and then classified according to taxonomy-centric metrics. The concern was sought to specify these defects so a more effective methods for DDBS performance improvement are set to be designed. The reduction of TC (including communication costs and response time) was the major motivators for which a good number of previous works sought to quench. Data locality maximization and data remote access mitigation were observed to be crucial issues that always need to be tackled wisely so TC is decreased.

Raouf et al. (2018) developed a cloud-based architecture for DDBS design. Data allocation for the resulted fragments along with the data replication at the run time were also considered so DDBSMs were able to work in parallel to process the queries of client's. The work had also studied the clustering of sites to further increase DDBS throughputs by maximizing locality of concerned data. Nevertheless, some drawbacks are recorded such as the selection of leaders of clusters which was intuitive and impractical to be considered in an efficient environment since almost most of DDBSs today have the same specifications for all sites, specifically in peer-two-peer network. In the meanwhile, Wiese et al. (2016) studied data replication problem (DRP) deeply that DRP was presented as an integer linear problem with the assumption of having the overlapping horizontally-split fragments. That is, the replication problem was treated as an optimization problem to gain the intended aim of having fragments' copies at the less number of sites of network. In the same line, Mahi et al. (2018) proposed a method based on Particle Swarm Optimization (PSO) algorithm to lessen TC through solving data allocation problem (DAP) by using PSO algorithm. Work's performance was observed and graded on 20 different test problems.

On the other hand (Sewisy et al., 2017; Amer, 2018), came to incorporate site clustering and the cost-effective model of data allocation and replication into one individual work. The results obtained were highly encouraging. Moreover, the authors in (Sewisy et al., 2017) drew the results in both cases, with site clustering and without site clustering to draw the impact of site clustering on DDBS performance. On the same page (Abdalla and Artoli, 2019), came to present an enhanced version of (Sewisy et al., 2017). This works was evaluated against (Sewisy et al., 2017) and shown to behave slightly better in most cases. A small-scale experimental study was conducted to exhibit the effectiveness of enhanced approach. A different data allocation scenarios were addressed, and the data replication was carried out using the replication model proposed in (Wiese et al., 2016). A significant enhancement was recorded in terms of overall DDBSs performance through TC mitigation. The constraints of clusters and sites were maintained to stimulate the real-world DDBS and tightened the proposed work's effectiveness.

Lastly, Luong et al. (2018) proposed k-Means rough clustering technique for vertical fragmentation. The distance and similarity were combined together with the upper and lower approximations to better proposed algorithm. The error average cost was seen to be high as both upper and lower approximations were addressed during the process of updating the new concentration.

### 2.2 Data allocation

In order for DAP problem to be solved, a good number of approaches were proposed in literature, and profoundly studied for both redundant and non-redundant states. In (Mukherjee, 2011), a cost model for data distribution over sites was presented to lessen communication costs. On the other hand (Tonini and Siqueira, 2013), came with an algorithm to find a distributed allocation schema so query performance was improved based on query history and data patterns analysis. A large biological database, as case study, was used for algorithm evaluation and promising results were observed.

However, static data allocation was consensually seen ineffectual in terms of DDBS performance in the ever-changing environment. So, to tackle this deficit, a well-structured approaches of dynamic nature are needed for data allocation in dynamic environment. A holistic data allocation approach was first proposed in (Apers, 1988), to find a solution for the problem of dynamically assigning data over network sites. In fact, this approach has been the core upon which many existing algorithms for dynamic data allocation have been built. For the same purpose, in Wolfson et al. (1997), a dynamic algorithm (named, adaptive data replication, ADR) was developed within a framework for dynamic data allocation. A genetic algorithm-based method was provided in (Rahmani et al., 2009) to solve data allocation problem in two steps. Firstly, site clusters was formed based on communication costs; secondly, the targeted data were scattered over clusters using GA.

By the same token (Singh, 2016), proposed a data allocation framework for non-replicated dynamic DDBS using the threshold and time constraint algorithms (TTCA). TTCA performance was experimentally compared with threshold algorithm on the basis of the total cost of reallocation and the number of fragment migrations over network. The findings illustrated that TTCA is more effective (in terms of performance promotion) than threshold algorithm chiefly as access frequency pattern changes swiftly. In (Kamali et al., 2011), an algorithm for tackling data allocation in replicated DDBS was evolved. Several aspects were included such as replication strategy and “non-uniform” distance between sites of network. The results obtained had shown that this algorithm yielded a good solution to data allocation in DDS. Some flaws, on the other hand, were noted like its being incapable of determining the number of fragment replica.

In the same line, a dynamic approach for fragment allocation was drawn in (Mukherjee, 2011). Time constraints, threshold value and the transmitted volume of data were taken into account. The problem of data allocation in the ever-changing environment was studied in (Li and Wong, 2013). Firstly, problem was defined and the use of time series models was enabled to perform short-term load forecasting. That is, the node number adjustment and fragment reallocation could be determined in advance. Subsequently, the nodes’ over-loadings and performance deterioration could be evaded particularly when fragment migrations is grown steadily. The load balancing was observed under the assumption that the future workloads can be modelled when time series was noted. In essence, the algorithm was presented to prove that the time series-based work outweighed threshold-based.

By the same token, a non-replicated data allocation approach was drawn in (Abdalla et al., 2014) in a dynamic environment. The given algorithm called “Performance Optimality Enhancement Algorithm” (POEA). It was destined to comprehensively integrate some concepts used in earlier algorithms. The time and sites constraints and the changing patterns of data access were taken into account. Moreover, the shortest path problem between sites was incorporated into POEA to be used when migration decision was being made. This step led to significant decrease in data migration. The experimental results draw a solid evidence that “POEA” had efficiently contributed in transmission costs and response time mitigation. Finally, to solve DAP problem (Lotfi, 2019), proposed a hybrid strategy using the differential evolution (DE) algorithm and variable neighborhood search (VNS) technique. The author sought to raise DE performance across the selection and crossover operators. The proposed approach aimed to navigate the search space via DE and performed further navigation using the neighborhood search technique. This approach was experimentally tested against state-of-the-art techniques and shown effective.

## 3 The proposed methodology

**Figure 1:**
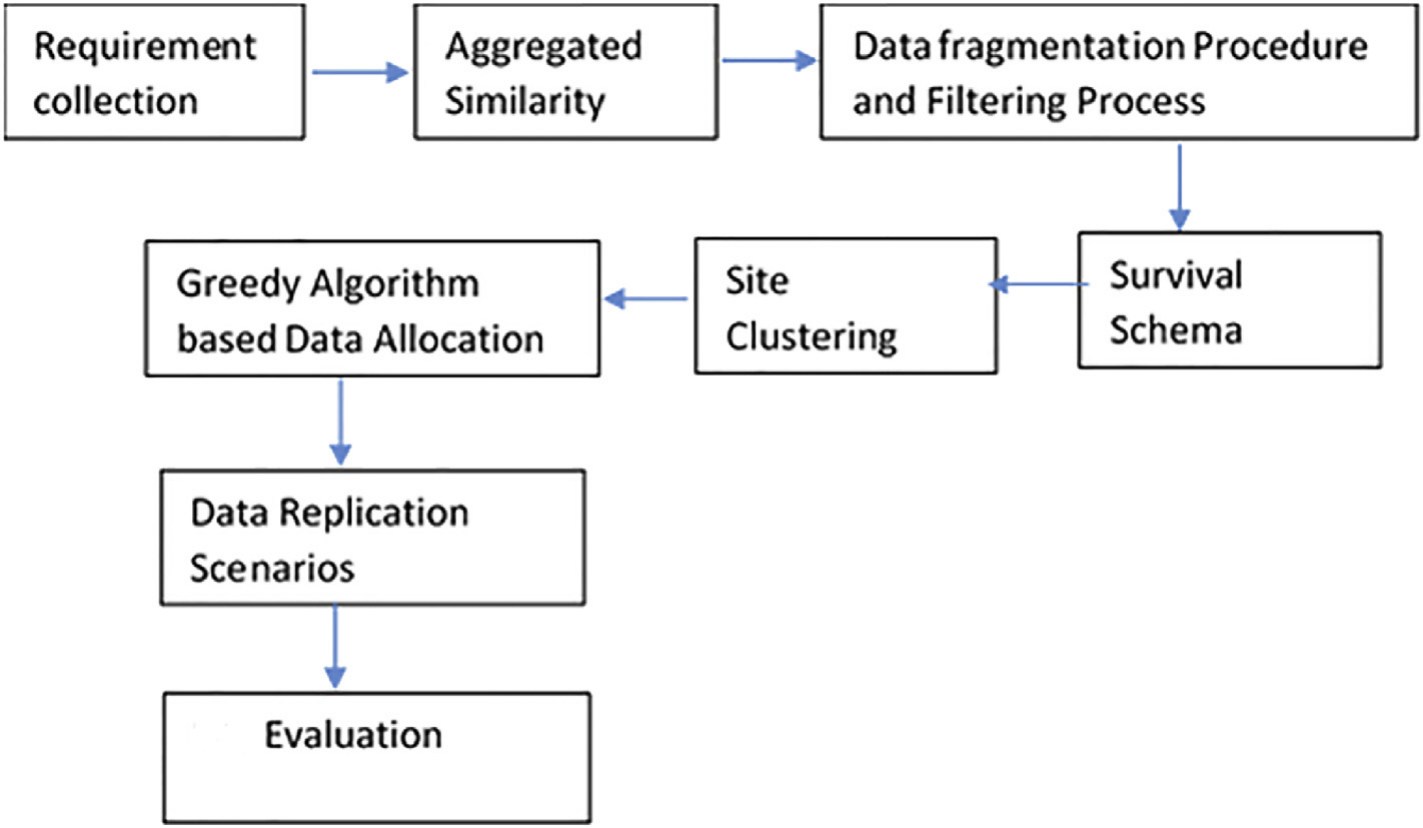
ASGOP diagram.

**Figure 2:**
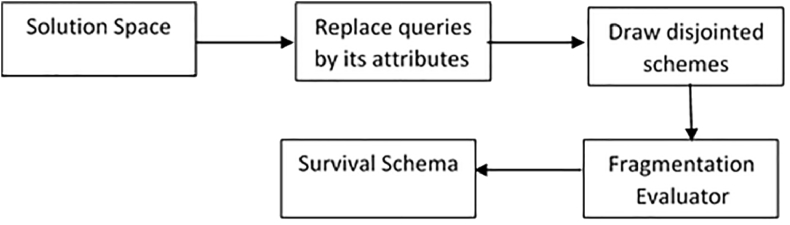
The schemes filtering process.

In this section we present our methodology for data fragmentation and allocation, as shown in Figures 1 and 2.

### 3.1 Data fragmentation procedure

The aggregated similarity measure is basically proposed to discover the hidden ties between queries. These ties would be effectively used to fragment database under consideration. Distance Matrix (DM) is found using the aggregated similarity and then passed into the agglomerative hierarchical clustering (AHC) process. The results of AHC, which are the overlapping clusters, would then be fed into the filtering process as depicted in Figure 2 to produce the non-overlapping schemes. After that, schemes would be moved into the proposed fragmentation evaluator (FE). FE (presented in section 3.5) seeks to find the survival schema based on the minimum remote access and maximum local access patterns. That is, the survival schema has the least remote access and the highest local access patterns. The overall process is visualized in Figure 1.

Figure 1 briefly exhibits the heuristics of ASGOP starting with collecting requirements (represented by dataset and queries). Then, the aggregated similarity function (see Eqn 5) would be directly applied on queries to provide distance matrix (similarity matrix in ASGOP). This matrix is then passed into AHC to provide data fragments (query clusters). Query clusters (as overlapping schemes) are set to be contained in a solution space. Space in its turn are placed into the filtering process (see Figure 2). The outputs of filtering process are the disjointed fragments which would be already drawn in the survival schema based on the results of fragmentation evaluator (see section 3.5.). After that, the sites of network is set to be clustered to yield cluster of sites and pave the way to starting process of data allocation. The final step is the approach evaluation which has been done in the discussion section below.

### 3.2 Similarity measure

The similarity measures have long been defined in literature and as the metrics using which how much alike two data points are set to be recognized. On the other hand, in the context of database, data mining, pattern recognition and almost all scientific fields, the similarity measure is used inversely as a distance (dis) between dimensions/features. Normally, if ‘dis’ is small, it is set to reflect the high degree of similarity. Conversely, if ‘dis’ is large, this similarity is low. Strictly speaking, similarity measure is basically contingent on the domain at hand. As a matter of fact, similarity has to be computed accurately when it is being considered across unrelated dimensions (features). Furthermore, the absolute values of similarity should be normalized (smoothed) to give the relative values so the dominance problem by features of higher values is eliminated. Simply put, similarity are set to be in the range [0, 1] as shown in Eq. (1).

(1) $$Sim\left(P_{i},P_{j}\right)=\;\{\begin{array}{c} V=1\;,\;\;\;\;\;\;\;\;\;\;\;\;\;\;\;\;P_{i}=P_{j}\\ 0<V<1,\;\;\;\;\;\;P_{i}∁\;P_{j};\;P_{j}∁\;P_{i}\\ 0,\;\;\;\;\;\;\;\;\;\;\;\;\;\;\;\;\;\;\;otherwise\; \end{array};\;P_{i}\cap \;P_{j}\;\neq \emptyset$$

Where P_i_ and P_j_ are two queries (represented as two points in the space of dimensions), i and j are just indices, and “V” is the value of similarity. In case (1), if both queries are equal, this means that these queries are equal, or one query is being duplicated. In case (2) either one query is being fully or partially contained within the other, their similarity is dependent on the degree of attributes containment. For example, if we have Q_1_ (A_1_, A_2_) and Q_2_ (A_1_, A_2_, A_6_), the value of V would be 2 out of 3, and Q1 is fully contained in Q2. Case (3), on the other hand, draw a zero similarity when both queries share non attribute. In this paper, thus, two similarity measures are being combined to produce an aggregated similarity as follows;

#### 3.2.1 Problem formulation

Provided that we have a set of “A” attributes A = {A_1_, A_2_, ..., A_n_} required by a set of “Q” queries, Qs = {Q_1_, Q_2_, ..., Q_q_}. For each query pair Q_i_ and Q_j_, both queries are treated as a string and the similarity measure is applied directly on attributes “As” of both queries. Following the fragmentation procedure described in section (3.1.), these queries would be already grouped into Cn clusters Cq_1_,Cq_2_…., Cq_cn_. These clusters would represent the overlapping data fragments, Fs = {F_1_, F_2_, …, F_fn_}.

#### 3.2.2 Hamming similarity measure (HS)

It has long been observed as an effective measure to find the match between strings. In its simplest definition, HS is computed using hamming distance (HD) which gives the number of shared digits between two string numbers (Hamming, 1950). For instance, given Q_1_ (1101) and Q_2_ (1010) as two queries represented by attribute existence in both queries (either “1” present attribute or “0” absent attribute) which are represented as string numbers; HD would have the difference value of (3) which will be then normalized by the number of all attributes (4 attributes) to give (3/4 = 0.75). However, as ASGOP seeks to find similarity, the similarity value of HD is (1–0.75) which is (0.25) because only the first digit in both numbers is being shared. Driven by this idea, this measure was combined in ASGOP with the nearby measure to produce an aggregated similarity measure which has been applied on queries directly. Queries are treated as strings and the major focus is to find the common attributes (seen as letters) between each query pair (seen as strings). The computation of HD as similarity measure is given in Eqs. (2) and (3). The similarity is sought to be firstly found using HD and is then aggregated with similarity value of nearby measure.

(2) $$Dif-Ham\left(Q_{i},Q_{j}\right)=\;\{\begin{array}{cc} Dis, & Q_{i}\;and\;Q_{j}\;are\;different\;queries\\ 0, & Otherwise \end{array}$$

Where ‘Dis’ represents the value of distance (difference) between different queries, and Q_i_ and Q_j_ are two different queries. However, since ASGOP seeks to find similarity, Eq. (3) is proposed.

(3) $$HS\left(Q_{i},Q_{j}\right)=\;1-\;\;\;\frac{Dif-Ham\;\left(Q_{i},Q_{j}\right)}{N_{a}}$$

‘Na’ in Eq. (3) is the number of all considered attributes, and is used as normalization factor as done in the given-above “toy” example.

#### 3.2.3 Nearby similarity measure (NM)

This measure is used to emphasize the similarity found by HD. In other words, whenever HD similarity value is found weak, NM would struggle to reinforce similarity between queries in two dimension space, Eq. (4).

(4) $$NM\left(Q_{i},Q_{j}\right)=\frac{\left(2\times \;Q_{ij}\right)}{\left(Q_{i}+\;Q_{j}\right)}$$

Where Q_i_, Q_j_ and Q_ij_ represents the number of attributes involved in both queries Q_i_ and Q_j_ severally, and the common attributes which are shared by both queries Q_i_ and Q_j_ respectively.

**Nearby measure example**: recall the same queries that are already drawn in HD example (Q_1_ and Q_2_), the value Q_ij_ is ‘2’ as both queries only share two attributes and the similarity measure is (4/6) which is 0.67.

#### 3.2.4 Aggregated similarity measure

As a combination of both HD and NM, the aggregated similarity is drawn in Eq. (5):

(5) $$Agg-Sim\;\left(Q_{i},Q_{j}\right)=\;0.5*HS\left(Q_{i},Q_{j}\right)+0.5*\;NM\;\left(Q_{i},Q_{j}\right)$$

Using this Equation, the optimal similarity is met if both queries were like each other, and similarity would have the value of “1”. Looking back at the given-above examples (1 and 2), the aggregated similarity is 0.74, which is still bigger than cosine measure (0.67), and have a reasonable “unexaggerated” maximization. Finally, we need to stress that the selection of weight (0.50) for each part of Eq. (5) is not an ad-hoc chosen. Essentially, we did process the aggregate similarity in three cases. Case (1): giving the first part a weight of 0.67 and the second part a 0.33 weight. Second case, giving first part a weight of 0.33 and second part a 0.67 weight. The third case was to give both weights the value of (0.50). The third case has been seen super competitive compared to the previous cases, and hence counted for our aggregated measure.

### 3.3 The hierarchical clustering (HC)

**Figure 3:**
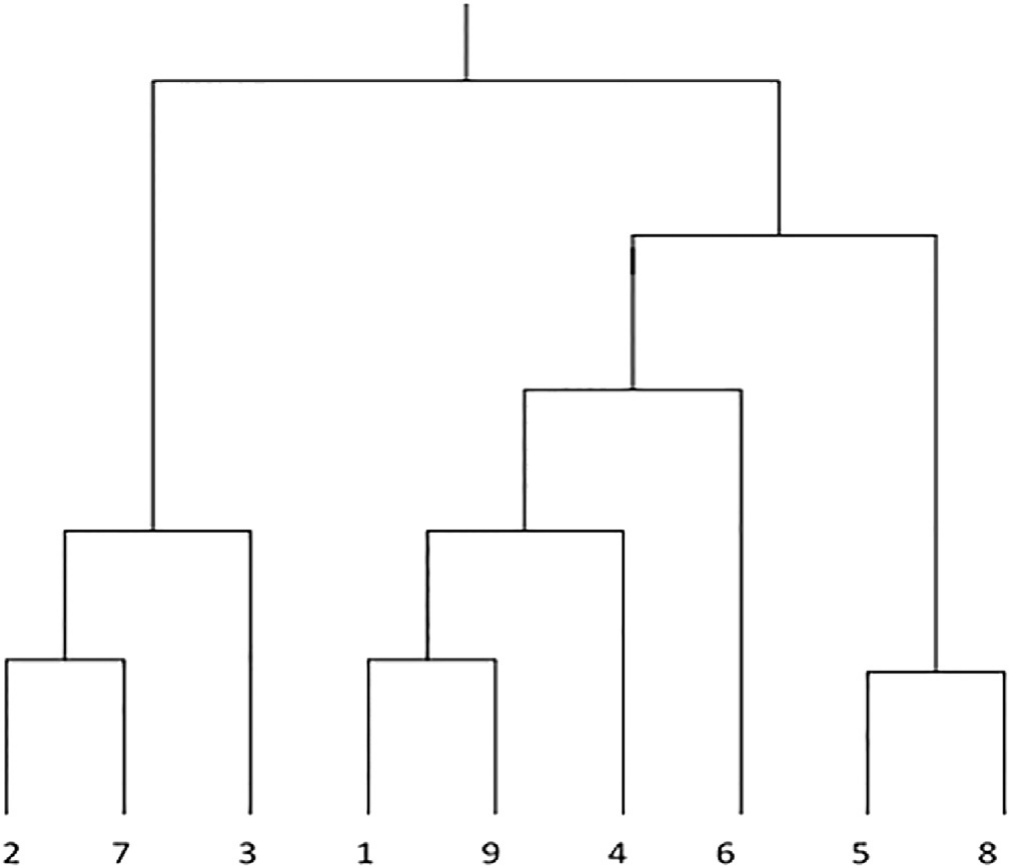
HC clustering Dendogram.

Generally speaking, HC aims at finding the nested sequence of clusters, with a single, all-inclusive cluster at the top and singleton clusters of individual items at the bottom. Then, each pair of clusters are combined in each intermediate level from the next lower grade or partitioning a cluster from the next higher grade. The result of a hierarchical clustering can be graphically drawn as tree, called a dendogram. Visually speaking, this dendogram depicts the combining process and the intermediate clusters. Figure 3 shows an instance of dendogram of nine points that were clustered into a single cluster. Furthermore, dendogram can provide a clear and easy query clustering and even a taxonomy, or hierarchical index. For HC approaches, there are two basic approaches to producing a hierarchical clustering:

(1) Agglomerative HC: it is the most widely used in literature. It starts with all data as single clusters and, at each successive step, the most similar or closest pair of clusters are combined together. The cluster similarity or distance definition is unavoidably required in order for the process to be completed.
(2) Divisive HC: starts with one, all-inclusive cluster and, at each successive step, the concerned cluster is constantly divided until only the singleton clusters of individual data are drawn. In each clustering step, it has to decide which cluster is going to be split and how the division will be performed.

In the proposed ASGOP, the single linkage agglomerative HC approach is used as follows: (1) the similarity between all pairs of data/points is computed, *i.e.*, compute the similarity matrix whose IJ^th^ cell refers to the similarity between the I^th^ and J^th^ points; (2) then, the most similar (closest) points are combined; (3) the similarity matrix is updated in each clustering step to reflect the new pairwise similarity between the new points and the original points; (4) finally, steps (2) and (3) are iterated till only a single cluster is found.

### 3.4 Query clustering

Our work comes in an attempt to eliminate the need to produce the numerical patterns of queries while ensuring the best design of DDBS. Out of saving computation time, the averaged distances between considered queries are computed directly as queries are processed as strings. In other words, the query numerical patterns that were presented in both (Sewisy et al., 2017; Abdalla and Artoli, 2019) are no longer needed. To compute the difference values between queries, the hamming distance metric (Eqn 3) and nearby measure (Eqn 4) have been used as a combination of both metrics to produce the aggregated similarity (Eqn 5) which resulted in the building of distance matrix. This matrix is then passed into AHC algorithm to create the initial overlapping schemes.

### 3.5 Fragmentation evaluator (FE)

FE consists of: 1. the Relevant Remote Access (RRM) which is the cost of access for remote attributes that are allocated at a site other than the site from which the concerned query is released (see Eqn 6); 2. The Irrelevant Local Access (ILA) which is concerned with attributes that are locally accessed (see Eqn 7). In FE evaluation, the lower FE result value is, the best DDBS performance is and vice versa.

(6) $$RRM=\;\overset{nf}{\underset{i=1}{\sum }}\overset{Q}{\underset{k=1}{\sum }}\left[FQ\;\left(Q\right)*\;\left|W_{ik}\right|\;\left(1-\;\frac{\left|W_{ik}\right|}{AN}\right)\right]$$

Where AN is the number of targeted attributes in the targeted fragment, K is an index for query set, I is an index for fragments, FQ is the frequency of query (how many times query is being released over network) that accesses data and W_ik_ is the number of attributes in fragment F_i_ which is locally accessed by Q_k_. Suppose that we have F (A_1_, A_3_, A_6_) and query Q (A_1_, A_2_, A_3_,A_5_) which has FQ of 10. Then, AN is 3 as F consist of three attributes. For the value of W_ik_, there has been two cases, if Q and F are existed in the same site, W_ik_ is 2 attributes (A_1_ and A_3_). Otherwise, W_ik_ in PRM Equation is zero as the query is a remote query with respect to F.

(7) $$ILA=\;\overset{nf}{\underset{i=1}{\sum }}\overset{Q}{\underset{k=1}{\sum }}\left[\overset{m}{\underset{j=1}{\sum }}FQ_{ki}^{i}*\;\left|Z_{ik}\right|*\left(\frac{\left|Z_{ik}\right|}{NA_{ijk}}\;\right)\right]$$

Where m is the number of remote sites, |Z_ik_| is the number of targeted A_k_ attribute(s) in fragment F_i_ which is remotely reached by Q_k_ with regard to the local fragment F_h_ which is already allocated in the same site (S_j_) of F_i_. NA_iqk_ is the entire number of attributes in fragment F_i_ distantly accessed, in regard to F_j_, by Q_k_. If we suppose query Q (A_1_, A_2_, A_3_, A_5_) is a remote query with respect to F (A_1_, A_3_, A_6_), then Z_ik_ would have a value of 2 attributes (A_1_ and A_3_) and NA_iqk_ has a value of 3 which is the number of all attributes of F. On the other hand, nf, Q and k are the number of considered fragments, queries, and attributes of queries respectively. I, q, and j are indices for these variables, though. Lastly, FE is being accumulated in Eq. (8) using these two components (PRM and ILA) as follows;

(8) FE=RRM+ILA

### 3.6 Site clustering

The presented algorithm of site clustering (See Algorithm 1) combines the nature of hierarchical clustering and the behaviour of the algorithm designed in (Amer et al., 2017). This process is mainly accomplished on the basis of the concept of Least Difference Value (LDV). LDV concept works similarly to the single-linkage hierarchical clustering. Each pair of sites has not been grouped in one cluster unless they have the minimum communication costs. According to the conducted evaluation, better results are satisfied in terms of TC reduction due to this combination.

Algorithm 1

**Table:** 

Input:	1.	Input: Network Sites; Communication Cost Matrix
	2.	Output: Site Clusters; Communication Cost Matrix between Clusters
	3.	Begin
	4.	Let m → the number of network sites; Cm→ the number of site clusters
	5.	Select all pairs of sites of the same lowest costs to be the newly-formed clusters
	6.	Initialize Cm→ the number of the newly-formed clusters
	7.	For all of these clusters, find their centeroid individually
	8.	Let Flag → True
	9.	For I → 1 to m
	10.	{ For J → 1 to Cm
	11.	If S(I) € C(J) let Flag → Flase
	12.	If Flag → True
	13.	{Let h → 2; k→ 0;
	14.	For J → 1 to C_m_
	15.	{If Distance (S(I), C(J).centeroid) < Distance (S(I), C(h).centeroid)
	16.	Mark S(I) to be added to C(J) and Let k → J
	17.	Else Mark S(I) to be added to C(h) and Let k → h
	18.	h++;}
	//to ensure that each site would join cluster/site of minimum cost, execute the follows:	
	19.	Let g →1; Flag-Site → True;
	20.	For s → 1 to m
	21.	If Distance (S(I), S(s)) < Distance (S(I), C(k).centeroid) and (I < > s)
	22.	{let Flag-Site → Flase; g = s;
	23.	Keep S(s).counter;}
	24.	If Flag-Site → Flase form new cluster (S(I),S(g))
	25.	Else add S(I) to C(k);
	}//if	
	26.	Go back to step 9
	}//For I	
Output:	Cluster of Sites

### 3.7 Data allocation strategy

To improve DDBS performance, data allocation must be carefully addressed with the basic aim of distributing data fragments into their relative clusters/sites from which they are constantly accessed. However, the complexity embedded in this procedure, due to the challenging mission of discovering the place for each data fragment, still impacts the overall performance profoundly. In ASGOP, therefore, a greedy oriented algorithm is proposed to find an acceptable solution. This algorithm seeks to minimize the objective function of the proposed model which is basically aimed at TC minimization. This function is crucially drawn with the aim of shrinking TC among network clusters. In this algorithm, each fragment would be tentatively given to each cluster/site and exposed on TC function at the same time. After that, the cluster/site with the least costs, depending on this function in terms of the targeted fragment, is the primary candidate to contain that fragment providing that cluster/site's constraints are preserved. Moreover, using this greedy algorithm, fragments are anticipated to be near-optimally allocated and replicated at the same time. That is, the algorithm strives to perform a simultaneous task represented in data allocation and replication on the fly. This algorithm is meant to have a high contribution with respect to DDBS performance promotion. Strictly speaking, the data allocation problem is treated as an optimization problem with the sole aim of further minimizing TC and rising performance. In other words, each fragment is allocated/replicated to cluster/site in which TC is kept at a minimum.

#### 3.7.1 Problem formulation

Provided that we have a set of “A” attributes A = {A_1_, A_2_, ..., A_n_} required by a set of “Q” queries, Qs = {Q_1_, Q_2_, ..., Q_q_}, and Qs is already grouped into Cn clusters {Cq_1_,Cq_2_…., Cq_cn_} using the proposed fragmentation technique. Then, the query clusters are scattered over a set of M sites S = {S_1_, S_2_, …., S_m_} which are also gathered into Cm clusters of sites {Cs_1_, Cs_2_, …., CS_cm_} in a fully connected network. The data allocation model primarily aims at finding the optimal distribution of each query cluster (Cq_i_) over clusters Cs_j_, and consequently over all sites of each concerned cluster. The optimal distribution is the case in which the minimum interaction between sites is obtained to answer distributed queries with the minimum TC.

#### 3.7.2 The proposed data allocation model (DAM)

The key idea of this model is that the data fragment should be assigned to the site in which the lowest TC is secured. This model led to a greedy strategy for data allocation and shown to perform well in practice through the empirical experiments. That is, using this model, fragments are allocated into their respective sites with the aim of obtaining the lowest interaction between sites of each cluster, and subsequently between clusters of the whole network. This objective led to obtaining the lowest Transmission Costs (TC) incurred over the entire network due to the distribution process of queries. The Objective function of this model is given by the next Eqs. (9), (10), and (11)

(9) $$TC\left(S_{j}\right)=\;Min\left(\overset{q}{\underset{k=1}{\sum }}\overset{m}{\underset{j=1}{\sum }}\overset{n}{\underset{f=1}{\sum }}FQ_{kf}*\;XF_{kj}*COM_{s_{i}s_{j}}*\left\{Sel\;\left(Q_{k}\right)*\;size\left(F_{f}\right)\right\}*M\right)\;,i=1,\ldots ,m$$

(10) $$TC\;\left(C_{h}\right)=\;\overset{m}{\underset{j=1}{\sum }}Min\;\left(TC\left(S_{j}\right)\right),\;\;m\;is\;the\;number\;of\;sites\;in\;the\;concerned\;cluster\;C_{h}$$

(11) $$TC\;\left(Network\right)=\;\overset{Cm}{\underset{h=1}{\sum }}Min\;\left(TC\;\left(C_{i}\right)\right),\;\;Cm\;is\;the\;number\;of\;network\;clusters$$

Where TC represents transmission costs that have been sought to be minimized while answering the distributed query, FQ is the total frequency of each released query and COM represents the communication costs between clusters/sites. This cost is either between sites (*i.e.* from site S_i_ to S_j_) or between clusters. The Sel (Q) is the selectivity percentage which refers to the data conveyed (actual data) by query as it has been answered. While the size of the fragment is referred to by Size (F), M indicates the number of sites which are involved in answering relative query. The smaller M value is, the smaller TC is, and vice versa. On the other hand, (k, j, h and f) are just indices to queries, sites, clusters, and fragments respectively. Finally XF is a binary variable indicates whether fragment allocated in the relative site (1) or not (0). While Eq. (9) seeks to minimize TC incurred over sites of each concerned cluster, Eqs. (10) and (11) struggle to accumulate these costs over each cluster and then over the whole network. This model is subjected to:

(12) $$Min\;\overset{Cn}{\underset{i=1}{\sum }}F_{i}$$

(13) $$\overset{\#fragments}{\underset{i=1}{\sum }}F_{ih}\geq 1\;\;\;\;h=1,\;\ldots ,\;Cm$$

(14) $$\overset{Cn}{\underset{h=1}{\sum }}C_{h}\;X_{hl}\;\leq \;C_{nfq},\;\;\;\;\;l=1,\;\ldots ,\;\#fragments$$

(15) $$\overset{m}{\underset{i=1}{\sum }}S_{j}\;Y_{il}\;\leq \;S_{nfq},\;\;\;j=1,..,m;\;l=1,\;\ldots ,\;\#fragments$$

(16) Xhi∈{0,1}h=1,…,Cn;i=1,…,#fragment

(17) Yil∈{0,1}i=1,…,#sites;i=1,…,#fragments

While Eq. (12) minimizes the fragment allocation in clusters/sites, Eq. (13) indicates that each fragment F_i_ must be allocated to all site clusters “Cm” in a replication scenario. In each cluster C_h_, fragment F_i_ was allowed to be replicated over several sites when it is needed. While nf and q refer to the number of fragments and queries already allocated in the concerned cluster/site respectively, Eqs. (14) and (15) ensure that cluster/site capacity has not been violated. In other words, Eqs. (14) and (15) enforce the capacity of each cluster/site as it must be kept unviolated. Finally, the variables (X and Y) are used as a binary (0, 1) in the last four Equations.

#### 3.7.3 The proposed greedy-based solution

To satisfy the desired reduction in TC, the data allocation solution addresses DAP based on the greedy -nature algorithm which is similar to the backpack problem so the objective function is optimized (minimized). The fragments represent the objects in the bag, and sites of the whole network represent the weights with respect to their accumulated TC incurred as each fragment is supposedly allocated into its relative site. The comparison process which involved examining the allocation of the intended fragment into the site based on TC is accomplished in the same procedure knapsack problem which is being solved, as given in the drawn-below illustrative example. Moreover, the intended fragment is replicated in the same process based on the calculated threshold. This model thus strives to simultaneously allocate and replicate each fragment into its respective site without extra complexity is being observed.

In its turn, a greedy algorithm always finds the solution that appears to be locally optimal at that time. This optimal-locality solution aims to find a globally-optimal solution. As mentioned earlier, the proposed model basically aimed at optimizing the drawn objective function by minimizing TC over the whole network on the basis of requirements given to the model. This optimization would be met through the greedy selection taken by dynamic programming represented in the knapsack-inspired algorithm. In fact, through experimental study drawn in this work, we found that this greedy algorithm elegant to be implemented and have a comparable results compared to the cost models (*i.e.* those models proposed in (Sewisy et al., 2017; Abdalla and Artoli, 2019)). Moreover, its run time more appealing and easier to be analyzed.

In ASGOP, on the other hand, the proposed solution has been working as follows: for each fragment (F_i_), F_i_ is listed along with its TC values over the considered sites of target clusters (C_j_). Those sites are given as input requirements to construct the dynamic programming table. This Table would handle F_i_ allocation process in the same way knapsack is being processed. After solving the knapsack-like table for the whole network clusters, the proposed model would extract a mini-matrix (Eqn 18) for only the concerned sites of the targeted cluster. This step is repeated each time F_i_ would be handled for allocation purpose over all clusters. In order for fragment to be replicated, a threshold value is computed based on values drawn in mini-matrix (see Eqn 19) and then the fragment would be replicated based on its TC on the relative sites of concerned cluster as drawn in Eq. (20). The final results of F_i_ would result in a simultaneous allocation and replication of fragments.

(18) $$Mini-Matrix\left(cluster,\;C_{i}\right)=\;\overset{m}{\underset{j=1,\;S_{j}\in C_{i}}{\sum }}\overset{Q}{\underset{k=1}{\sum }}Max-Matrix\left(S_{j},F_{k}\right)$$

Where m and Q stand for the number of sites and queries over the whole network. This Equation draws the mini-matrix of each cluster which is basically extracted from Max-Matrix. The Max-Matrix is the matrix that contains the whole network information with respect to transmission costs (see Table 2, below).

(19) $$THV=\frac{\left(Max\;\left(mini_{matrix\left(i,j\right)}\right)+Min\;\left(mini_{matrix\left(i,j\right)}\right)\right)}{2}$$

(20) $$AR-Decision\;\left(Fragment\right)=\{\begin{array}{cc} 1, & TC\;\left(Fragment\right)\leq \;THV\\ 0, & Otherwise \end{array}$$

Where both the Max (mini-matrix) and the Min (mini-matrix) are taken from Max-Matrix (see Table 2) as the maximum and minimum values of the matrix. On the other hand, the THV and AR-Decision stand for threshold value and fragment allocation and replication decision respectively. Finally, i and j are just indices. It is worth indicating that when TC of the respective fragment is less than THV that means fragment is being allocated/replicated in cluster/site in which TC is being reduced to a minimum, and vice versa.

#### 3.7.4 Data allocation algorithms

Algorithm 2

**Table:** 

Input:	**The most-used** Query List; Query original site List (sites from which queries are most released); Cluster communication cost matrix; Site communication cost matrix; Selectivity matrix; Fragments information, Fragment number, Cluster number, Sites, FSTC.	
	**Begin**	
	1.	{
	2.	For (f = 0 to Fn) V [0,f] = 0;
		//Fn number of fragment; V refers to value of TC for each fragment f. when there
		is no f in respective site, value is 0.//
		//steps 4–13 to draw Max-Matrix (see Table 2)
	3.	For (i = 1 to m)//number of sites of the whole network
	4.	For (f = 0 to F)//number of fragment
	5.	If ((f [i] <=f) and (v [i]+V [i-1, f-f [i]] > V [i-1,f]))
	6.	{V [i,f] = v [i] + V [i-1, f-f [i]]];
	7.	Keep [i,f] = 1;
	8.	}
	9.	else
	10.	{V [i,f] = V [i-1,f];
	11.	Keep [i,f] = 0;
	12.	}
	13.	K=F
	14.	For (i = m down to 1)//backtracking to retrieve sites in which TC is produced
	15.	If (keep [I,k] = = 1)
	16.	{Output I;
	17.	K=K-f [i]
	18.	}
	19.	Return V [m,F]
		//M is the number of returned sites in target cluster that will be holding F list.
	}//**End**	
Output:	Fragments Allocation over Clusters

Algorithm 3

**Table:** 

Input:	**Begin**	
	
	1.	For I → 1 to F_n_//number of fragments
	2.	For K → 1 to C_m_//Cm is number of clusters
	3.	{Temporarily allocate fragment F(I) into site (1) of cluster K C(k). S (1);
		//S (1) is the first site in Cluster (k)
	4.	For J → 2 to m//m number of sites in cluster C(k)
		//we start from site (2) as site (1) is already buffered with F(I) in step 3
	5.	{Temporarily re-allocate F(I) into S(J);
	6.	If TC (F(I), S(J)) < TC (F(I), S (1));//to find site of minimum TC
		//step 6: to find which site has the minimum TC in regard to F(I)
	7.	let H → J
	8.	Else let H → 1;
		//H is index to the survival site of minimum TC
	9.	}//For J
	10.	Permanently allocate (F(I) into S(H));
		//allocate F into site of minimum TC
	11.	}//for Cn
	12.	Go back For I loop
	13.	}//for I
	
	**End**	
	
Output:		Fragments Allocation over Sites in each Cluster

**Figure 4:**
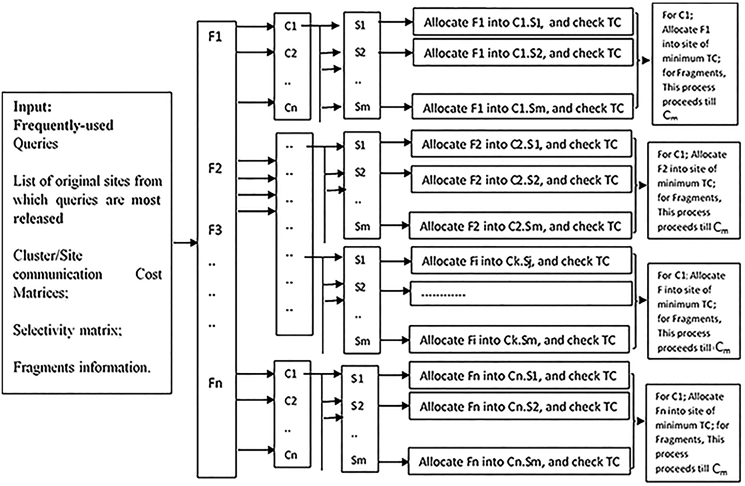
Abstract level view of data allocation process.

Moreover, as an abstract flow diagram, Figure 4 further clarifies all have-to-be-done steps of data allocation of the proposed work.

#### 3.7.5 Illustrative example for the mechanism of greedy-based algorithm

Assuming that we have nine sites that are already clustered using the site clustering algorithm proposed in section (3.6.). Suppose that Cluster C_1_ consists of (S_1_, S_5_, S_3_, and S_4_) and TC values of fragment F_1_ are listed along with each respective site as drawn in Table 1. These values are drawn based on computing TC function given in section (3.7.2.) and F_1_ is randomly allocated to each site once. In each random allocation, F_1_ is being allocated to a different site so its’ TC is examined to record the site(s) in which F_1_ yields the minimum TC.

**Table 1:** Fragment F1 list along with its TC values over considered sites of C1.

Fragment	TC	Site
F_1_	12%	S1
F_1_	8%	S5
F_1_	7%	S3
F_1_	4%	S4

Using Table 1 as input requirement, Table 2 as Max-Matrix is constructed to run dynamic programming (as described in section 3.7.4.) on “F_1_“ list so the most suitable sites for F_1_ are selected based on the minimum interaction between each site in C_1_ and the whole network.

**Table 2:** Max-Matrix for fragment F1 allocation.

S		S_1_	S_2_	S_3_	S_4_	S_5_	S_6_	S_7_	S_8_	S_9_
F	0	0	0	0	0	0	0	0	0	0
F_1_	1	12	12	12	12	12	12	12	12	12
F_1_	2	12	12	12	12	12	20	20	20	20
F_1_	3	12	12	12	19	19	20	20	20	27
F_1_	4	12	12	12	19	19	20	20	23	27

After that, the matrix of concerned cluster (contains only sites of cluster, C_1_) would be extracted from the whole matrix of the network (Max-Matrix). The extracted matrix, called mini-matrix, is drawn in Table 3.

**Table 3:** Mini-matrix of F_1_.

S		S1	S3	S4	S5
F	0	0	0	0	0
F_1_	1	**12**	12	12	12
F_1_	2	12	12	12	12
F_1_	3	12	12	19	19
F_1_	4	12	12	19	**19**

To avoid both the challenge in estimating threshold values by the user and the implication resulted in implementation difficulty, the threshold is loosened to be a problem-centric (data-defined) threshold. By taking threshold based on Eq. (19) and Table 3, the threshold value is [(12 + 19)/2 = 16] in this example. That is because of that the value of (12) is the minimum value and the value of (19) is the maximum value in Mini-Matrix. We find that F_1_ shall be allocated and replicated in S_1_ and S_3_ only as they produced the minimum averaged TC (minimum interaction) over cluster C_1_. In other words, their TC did not exceed threshold value which is (16). On the other hand, S_4_ and S_5_ are being excluded as they have conflict values. While the first two rows indicate that both sites have the value of (12), the last two rows indicate that both sites have the value of (19) which also exceeded threshold value. The value of TC of each site in regard to the concerned fragment must be decisive (always less than or equal threshold) so fragment would be considered for site allocation. This process is set to be repeated in each cluster for each fragment to be simultaneously allocated and replicated.

## 4 Results

In this section we are going to: present experimental setup for ASGOP and its competitive peers, draw the datasets used in experiments conduction, give a demonstrative example to show the way in which ASGOP works, and finally, analyze ASGOP performance along with its peers.

### 4.1 Experimental setup

**Figure 5:**
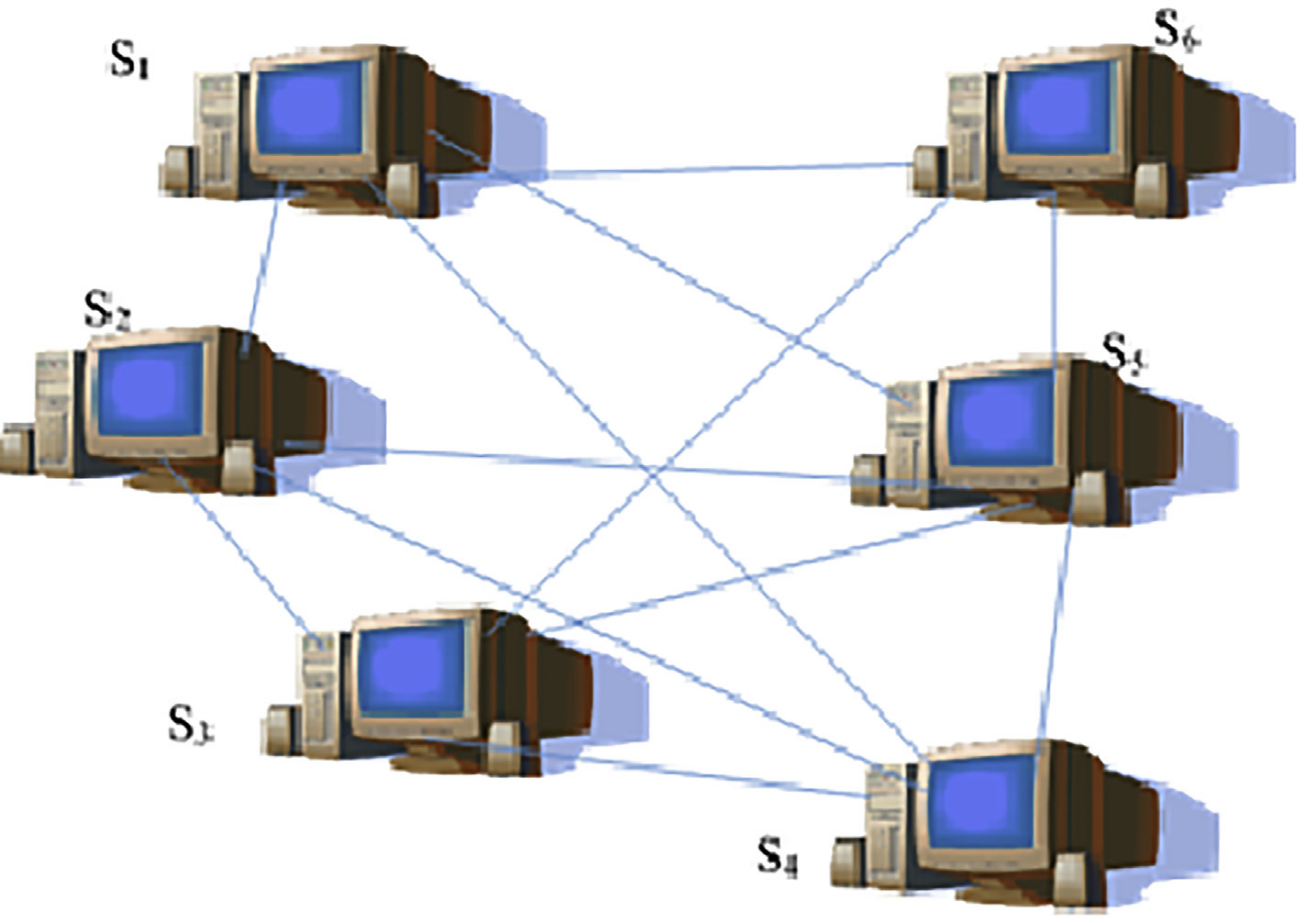
Network sites.

This work has been executed using a C++ programming language which runs on a processor of 1.7 GHz Intel (R) Dual-Core (TM) i3CPU with 2 GB of main memory and 80-GB hard drive. All requirements including queries and their frequencies over sites are assumed to be gathered from DDBS workload. The first experiment is exclusively conducted in an attempt to demonstrate ASGOP mechanism. On the other hand, performance evaluation is being carried out internally, and externally with (Sewisy et al., 2017; Abdalla and Artoli, 2019) to show ASGOP supremacy. The virtual network is assumed to be fully-connected of six sites, as shown in Figure 5.

### 4.2 Datasets

#### 4.2.1 Ships dataset

Table 4 reveals the dataset description of the synthetically-proposed ships (with 400 records) in the first experiment for illustration purposes. In the evaluation section, dataset records have been diversified over several experiments.

**Table 4:** Ships database description.

Attributes	Symbol	Type	Length (Bytes)
Ship-no	A_1_	Nominal	4
Ship-name	A_2_	Categorical	30
Captain-id	A_3_	Categorical	4
Captain-Wage	A_4_	Numerical	3
Port	A_5_	Categorical	5
Port-id	A_6_	Nominal	4

On this dataset, for demonstration purposes, eight queries are identified in the first experiment as the most frequently running queries. While seven queries were supposed to be of retrieval type, one single query was supposed to be of update type. This was drawn intentionally to diversify the rate of queries from the first experiments. This diversity would be varied through all experiments. Actually, this step is meant to show the impact of query type on DDBS performance.

Q_1_: Select A_1_, A_3_, A_5_ from Ships where A_1_ in (1234, 261, 1239) and A_3_ = “M222”;

Q2: Select A_1_, A_2_, A_5_, A_6_ from Ships where A_5_ in (‘site 1’, ‘site 3’, ‘site 6’);

Q3: Select A_2_, A_3_, A_5_ from Ships;

Q4: Select A_1_, A_4_, A_6_ from Ships where A_6_ = ‘dept2’;

Q5: Select A_2_, A_4_, A_5_ from Ships where A_2_ = “Jane” and A_5_ in (‘site 2’,‘site 5’);

Q6: Select A_2_, A_3_, A_4_, A_6_ from Ships where A_4_ > 4500;

Q7: Update Ships set A_2_ = ‘Ali’, A_6_ = 2 where A_1_>1234;

Q8: Select A_1_, A_3_, A_5_ from Ships;

#### 4.2.2 Employee dataset

For further analysis and discussion for ASGOP performance and its peers, the employee dataset is proposed based on the description drawn in Table 5 which is basically taken from (Abdalla and Artoli, 2019). This dataset is mainly used to ensure a fair comparison for ASGOP with its peers. This dataset has six attributes and filled out with records ranges from 300 to 1200 records over all experiments as given in Table 16.

**Table 5:** Employee database.

Attributes	Symbol	Type	Length (Bytes)
Emp-no	A1	Nominal	4
Emp-name	A2	Categorical	30
Job-id	A3	Categorical	4
Salary	A4	Numerical	3
Loaction	A5	Categorical	5
Dept-id	A6	Nominal	4

Furthermore, for the first problem, it is supposed that we have eight queries under consideration as the sample for the most frequently-running queries against the Employee dataset.

Q1: Select A_2_, A_4_, A_6_ from Employee where A_4_ between (1500, 3000);

Q2: Select A_1_, A_5_ from Employee where A_5_ in (‘site 2’, ‘site 3’);

Q3: Select A_1_, A_3_, A_6_ from Employee;

Q4: Select A_1_, A_2_, A_3_ from Employee where A_3_ = ‘manager’;

Q5: Select A_3_, A_4_, A_5_ from Employee where A_3_ = “worker” and A_4_ > 1200;

Q6: Select A_2_, A_4_, A_5_ from Employee where A_4_ < 2500;

Q7: Select A_3_, A_6_ from Employee where A_6_ = ‘dept2’;

Q8: Select A_1_, A_4_, A_5_ from Employee;

### 4.3 Running example

In this example, using the listed-above queries in section (4.2.1.), the behavior of ASGOP has been illustrated step by step along with the example that is already given in section (3.7.5). First of all, the Query Attribute Incidence Matrix (QAIM) of the first experiment was formed in Table 6.

**Table 6:** Query attribute incidence matrix (QAIM).

Q/A	A1	A2	A3	A4	A5	A6
Q1	1	0	1	0	1	0
Q2	1	1	0	0	1	1
Q3	0	1	1	0	1	0
Q4	1	0	0	1	0	1
Q5	0	1	0	1	1	0
Q6	0	1	1	1	0	1
Q7	1	1	0	0	0	1
Q8	1	0	1	0	1	0

After that, using the procedure of fragmentation that was drawn in section (3.2), all these requirements are fed into the fragmentation procedure including hierarchical clustering Process (HC), refinement process and fragmentation evaluator (FE) on the proposed dataset of Ships. By applying Eqs. (2), (3), (4), and (5) on QAIM, Tables 7, 8, 9 holds the values of these Equations.

**Table 7:** Hamming based similarity matrix.

Q/A	Q1	Q2	Q3	Q4	Q5	Q6	Q7	Q8
Q1	1.00	0.50	0.67	0.67	0.33	0.17	0.33	1.00
Q2	0.50	1.00	0.50	0.50	0.50	0.33	0.83	0.50
Q3	0.67	0.50	1.00	0.00	0.67	0.50	0.33	0.67
Q4	0.33	0.50	0.00	1.00	0.33	0.50	0.67	0.33
Q5	0.33	0.50	0.67	0.33	1.00	0.50	0.33	0.33
Q6	0.17	0.33	0.50	0.50	0.50	1.00	0.50	0.17
Q7	0.33	0.83	0.33	0.67	0.33	0.50	1.00	0.33
Q8	1.00	0.50	0.67	0.33	0.33	0.17	0.33	1.00

**Table 8:** Nearby similarity matrix.

Q/A	Q1	Q2	Q3	Q4	Q5	Q6	Q7	Q8
Q1	1.00	0.57	0.67	0.67	0.33	0.29	0.33	1.00
Q2	0.57	1.00	0.57	0.57	0.57	0.50	0.86	0.57
Q3	0.67	0.57	1.00	0.00	0.67	0.57	0.33	0.67
Q4	0.33	0.57	0.00	1.00	0.33	0.57	0.67	0.33
Q5	0.33	0.57	0.67	0.33	1.00	0.57	0.33	0.33
Q6	0.29	0.50	0.57	0.57	0.57	1.00	0.57	0.29
Q7	0.33	0.86	0.33	0.67	0.33	0.57	1.00	0.33
Q8	1.00	0.57	0.67	0.33	0.33	0.29	0.33	1.00

**Table 9:** The aggregated similarity matrix.

Q/A	Q1	Q2	Q3	Q4	Q5	Q6	Q7	Q8
Q1	1.00	0.54	0.67	0.67	0.33	0.23	0.33	1.00
Q2	0.54	1.00	0.54	0.54	0.54	0.42	0.85	0.54
Q3	0.67	0.54	1.00	0.00	0.67	0.54	0.33	0.67
Q4	0.33	0.54	0.00	1.00	0.33	0.54	0.67	0.33
Q5	0.33	0.54	0.67	0.33	1.00	0.54	0.33	0.33
Q6	0.23	0.42	0.54	0.54	0.54	1.00	0.54	0.23
Q7	0.33	0.85	0.33	0.67	0.33	0.54	1.00	0.33
Q8	1.00	0.54	0.67	0.33	0.33	0.23	0.33	1.00

Table 9 is then fed into AHC to produce the final results (Clusters) and find solution space (Table 10).

**Table 10:** Solution space.

Solution #	Cluster #	Queries contained
Solution 1	Cq1	Q1573 Q2468
Solution 2	Cq2	Q1235 Q4678
Solution 3	Cq3	Q2578 Q1346
Solution 4	Cq4	Q13567 Q248

This space was set to be passed into the filtering process to remove attributes overlapping between the obtained schemes (as each query would be replaced by its contained attributes). The results were the disjointed schemes. By following the same procedure described in section (3.2), the final schema is represented in Table 11.

**Table 11:** Survival schema.

Fragments	F1	F2
Contents	A_1_,A_2_,A_4_,A_5_,A_6_	A_3_
Size in Byte	18800	1600

#### 4.3.1 Allocation process

As mention earlier, fragments allocation is made in such a way that guarantees the allocation of each fragment to its perfect destination in keeping with TC minimalism. In the sense that based on the greedy-based data allocation algorithm of section (4), each fragment would be temporarily placed into each site and exposed on TC at the same time. Whenever TC has been purely minimized as a result of fragment placement on the concerned site, the fragment is therefore permanently assigned to that site. According to evaluation results, this kind of data allocation contributed to lessening TC to a great extent as the distributed query was being processed. On the other hand, according to (Sewisy et al., 2017; Abdalla and Artoli, 2019), with which we draw an external evaluation, the best scenarios proved to be the best-fitting solution for DDBS design are replication and non-replication based scenarios. Consequently, the fragments allocation algorithm of ASGOP was made in these two scenarios as well. While the first scenario assumed that each fragment would be replicated over all clusters of sites; the second scenario assigned each fragment to a single cluster/site of minimum TC. Such a procedure of data allocation scenario made ASGOP comparable with all considered works in terms of both TC and DDBS performance, as shown in the evaluation section.

#### 4.3.2 The first allocation scenario (fragments replicated over cluster of sites)

**Phase1**: Fragments are directly allocated to all clusters using replication principle as shown in Table 12.

**Table 12:** Data fragment allocation (final step).

Cluster	C1				C2	
Fragment/Sites	S1	S2	S4	S6	S3	S5
F1	398090	376940	**242990**	306440	**271190**	297510
F2	41100	41100	**15600**	35100	13800	**13200**

**Phase** 2: in each cluster, following the results of the greedy based algorithm, each fragment is assigned to site that satisfies TC minimalism, Table 13. The final allocation of data fragments (including replica) over clusters and sites is drawn in Table 13.

**Table 13:** Final allocation map.

Cluster	C1				C2	
Fragment/Sites	S1	S2	S4	S6	S3	S5
F1			1		1	
F2			1			1

From Table 12, by comparing TC measures of all sites for each cluster, it is clear that F_1_ was assigned to S_4_ in C_1_ and S_3_ in C_2_. While F_2_ is allocated to S_4_ of C_1_ and S_5_ of C_2_ respectively (Table 13).

#### 4.3.3 The second allocation scenario (no fragment replication)

**In this one-phase scenario,** each fragment is placed into the site of minimum TC based on the proposed algorithm as shown in Tables 14, 15.

**Table 14:** Data fragment allocation (final step).

Cluster	C1				C2	
Fragment/Sites	S1	S2	S4	S6	S3	S5
F1	398090	376940	**242990**	306440	271190	297510
F2	41100	41100	15600	35100	13800	**13200**

**Table 15:** Final allocation map.

Cluster	C1				C2	
Fragment/Sites	S1	S2	S4	S6	S3	S5
F1			1			
F2						1

The final allocation for fragments is drawn in Table 15.

It is worth referring that we just drew the results of the final steps of proposed greedy-based algorithm. That is, Tables 12, 14 are the final outcomes.

### 4.4 Performance evaluation

In essence, like its counterparts, ASGOP comes in an attempt to improve DDBS performance through data locality maximization to a great possible extent as each fragment is given to the site from which it is frequently required. Moreover, the proposed data allocation procedure has been designed on the basis of greedy nature that tacitly guaranteed to minimize the network overheads and fragments migration. As a result of such design, TC has strongly been believed to be significantly reduced and DDBS productivity to be steadily increased. In a solid step to verify these claims, an internal and external evaluation has been made. Five experiments have been addressed, each of which constitutes of several problems and each problem has different requirements. For the first experiment, just the first problem (with eight queries and six sites) has exclusively been investigated in section (4.3). The next problems, for all experiments, are treated in the same manner the first problem was being processed.

**Figure 6:**
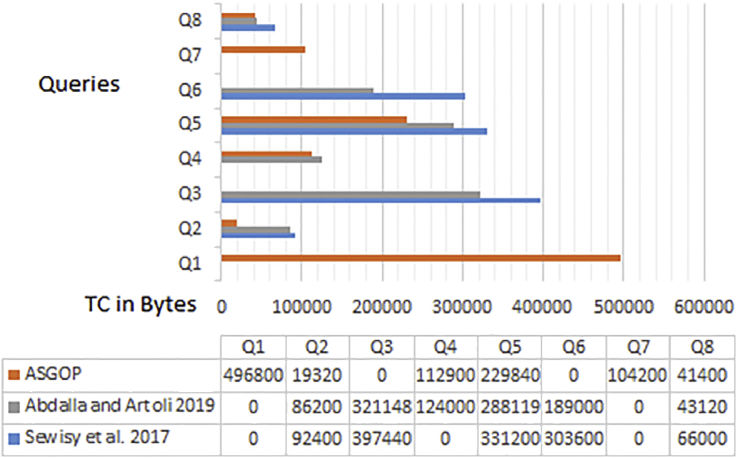
Replication scenario- TC

**Figure 7:**
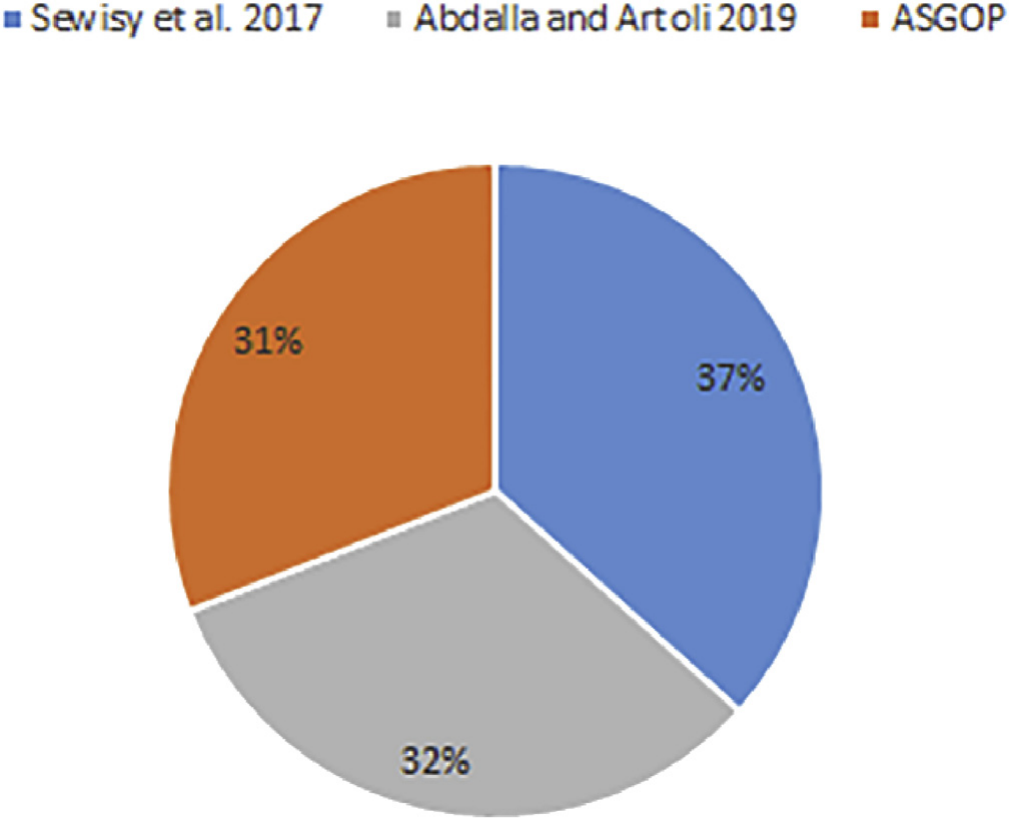
Average of TC - replication scenario.

**Figure 8:**
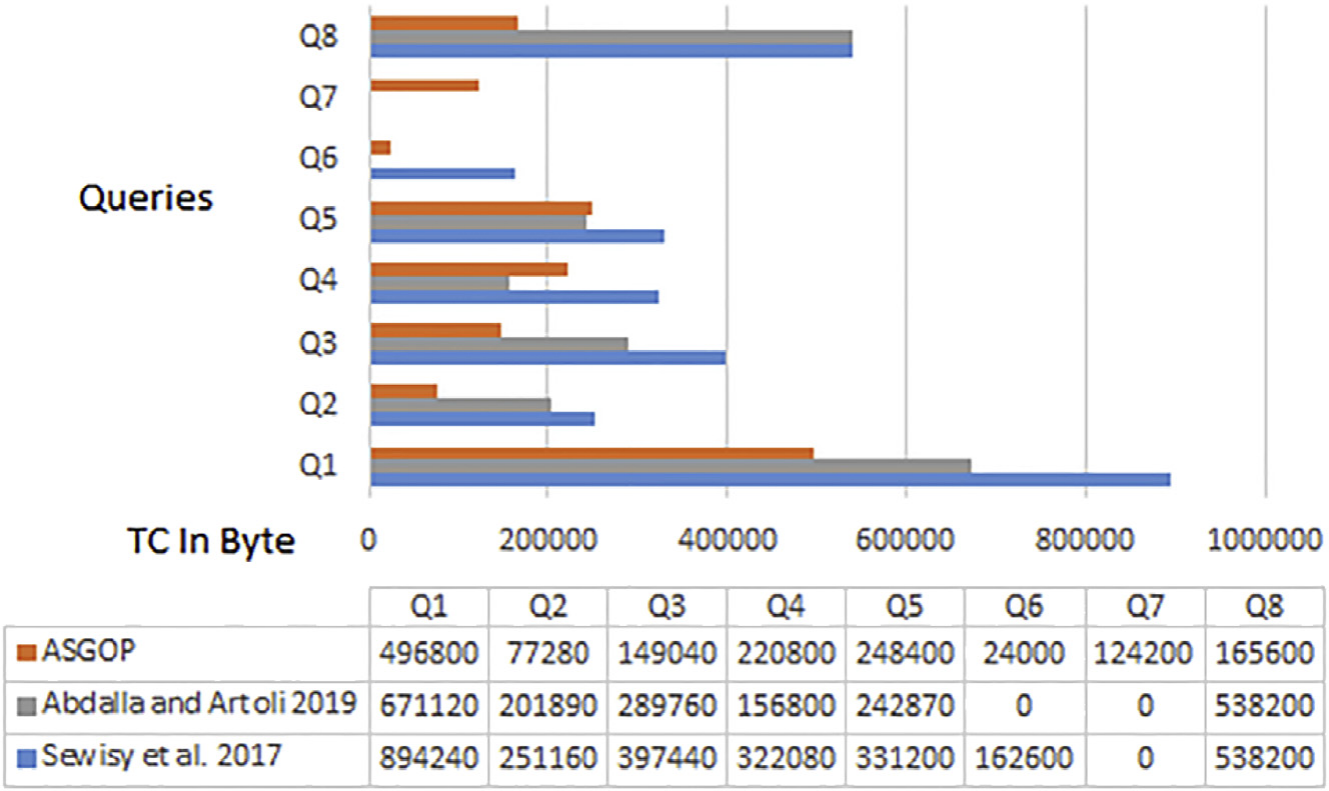
Non-replication scenario - TC

**Figure 9:**
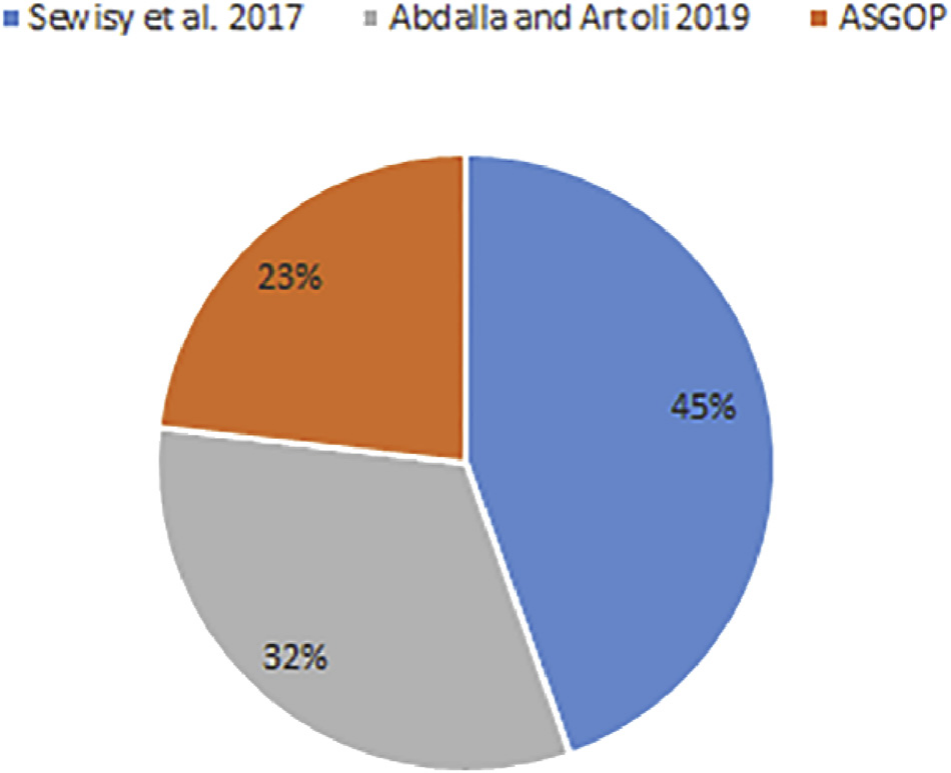
The average of TC - non replication scenario.

Simply put, for the first problem of first experiments (shown in Figures 6, 7, 8, and 9), every query, among those under consideration, was separately tested on the given dataset of ships in accordance to two scenarios: **1**. allocation scenario-1 in which all fragment were replicated over all clusters of sites; **2**. allocation scenario-2 in which fragments were allocated to sites of minimum TC values regardless of their clusters. On the other hand, as the closest works for ASGOP (Sewisy et al., 2017; Abdalla and Artoli, 2019), were involved in a competitive comparative process. All of these experiments have been conducted for all works: ASGOP (Sewisy et al., 2017), and (Abdalla and Artoli, 2019). To be in parallel with experiments that were drawn in (Sewisy et al., 2017) and (Abdalla and Artoli, 2019), all problems of all experiments considered a mixture of retrieval and update queries so that retrieval queries started to occupy large space from the first problem, and this space was being gradually reduced from one problem to the next problem in the favor of update queries. Such diversity in queries type was being purposefully done for the purpose of verifying technique's performance under several circumstances. The last two problems of the experiment were also a mixture of retrieval and update queries with update ones occupying larger space. Finally, for each problem, the minimization of (TC) has been monitored and recorded.

From Figure 6 which depicts the results of the first problem's processed queries; it can be said that ASGOP (Sewisy et al., 2017), and (Abdalla and Artoli, 2019) were alternately behaving better in terms of replication-based scenario with ASGOP being slightly superior (Abdalla and Artoli, 2019). behaved slightly better than (Sewisy et al., 2017), though. In other words, no significant superiority was observed for either one of them. However, when results are taken in total, Figure 7 showed that ASGOP has a slight improvement in regard to TC minimization although all works were still close to each other. It is worth indicating that zero values of query execution costs in Figures 6 and 8 mean that this query is accessed locally leading, as a result, to have a zero value for TC which is essentially dominated by the remote access.

According to results obtained in Figure 6, ASGOP outperformed its counterparts with respect to Q_2_, Q_3_, Q_5_, Q_6_, and Q_8_. For Q_4_ (Sewisy et al., 2017), outweighed ASGOP significantly, though. However, ASGOP recorded the worst results in Q_1_ and Q_7_ for the next reasons. For Q_1_, the number of sites involved in processing query was bigger than it was in ASGOP's counterparts. On the other hand, due to the fact that Q_7_ is of update type and allocation scenario is the replication-based, ASGOP recorded worst results in this scenario as updated data needs to be replicated in each site into where this data was already stored. In general, ASGOP does not work in the replication-based allocation scenario as good as it is drawn in the non-replication allocation scenario.

On the other hand, when it comes to the non-replication scenario in Figure 8, ASGOP demonstrated its superiority over both (Sewisy et al., 2017; Abdalla and Artoli, 2019) with the sole exception recorded for query7. Also (Abdalla and Artoli, 2019), still behaved much better than (Sewisy et al., 2017). More evidently, as results would also be considered in total, the final results were also in favor of ASGOP as depicted in Figure 9.

**Figure 10:**
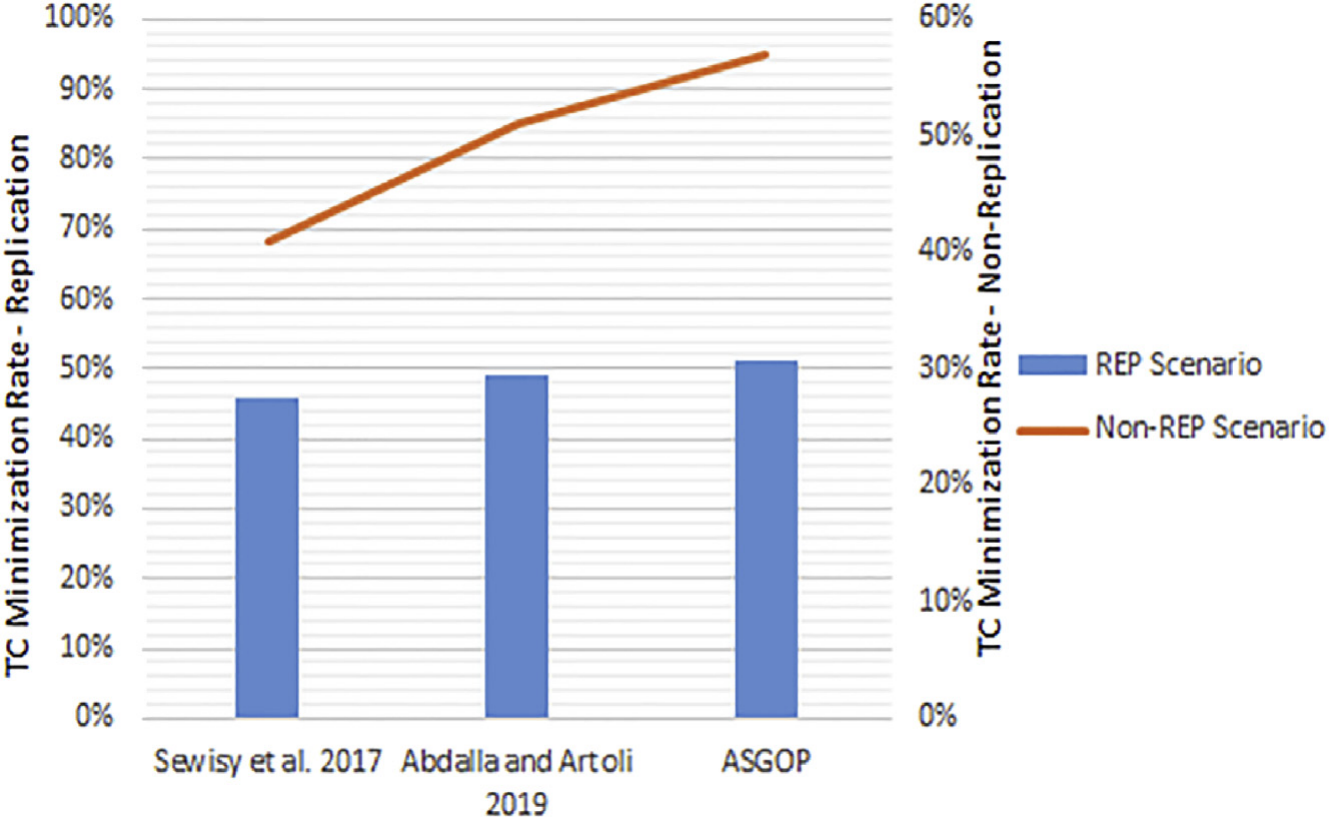
DDBS Performance- The first problem.

Finally, for problem (1) of the first experiment, as a reflection of Figures 7 and 9 that depicted TC, Figure 10 sought to clearly visualize DDBS performance of all works in terms of both the replication and the non-replication scenarios. For replication scenario, ASGOP showed a slight difference followed by (Abdalla and Artoli, 2019). However, ASGOP was proven to be more effective for the non-replicated scenario. It is worth indicating that axis y which is given the name “TC” in Figure 10 refers to the minimization rate of TC each approach satisfied.

### 4.5 Employee dataset

To affirm the performance of ASGOP under different circumstances for both scenarios, further experiments on employee dataset (dataset description along with a sample of eight queries used which are already given in section 4.2.2) have also been conducted within the frame of the first experiment (the number of sites still six sites). It is worth indicating that the extended comparison has been restricted to be between ASGOP and work of (Abdalla and Artoli, 2019). That is due to the fact that the experiments that were drawn in (Abdalla and Artoli, 2019) provided that (Abdalla and Artoli, 2019) had outweighed (Sewisy et al., 2017) substantially. Moreover, according to the first experiment of this paper (see Figures 6, 7, 8, 9, and 10), both ASGOP and (Abdalla and Artoli, 2019) are superior to (Sewisy et al., 2017). Given these facts and out of saving computation time (Sewisy et al., 2017), is being excluded from any further examination. On the other hand, each problem (among all problems addressed in Table 16) has different requirements form one to another in terms of the number of records and queries under consideration as well as the percentage of query-type space.

**Table 16:** Problems requirements.

#sites = 6				
P#	Q#	Record#	Q-Read%	Q-Update%
P1	8	300	100%	0%
P2	16	500	87.50%	12.50%
P3	24	650	85.00%	15.00%
P4	30	650	82.00%	18.00%
P5	36	800	82.00%	18.00%
P6	42	1100	80.00%	20.00%
P7	45	1200	75%	25%

**Figure 11:**
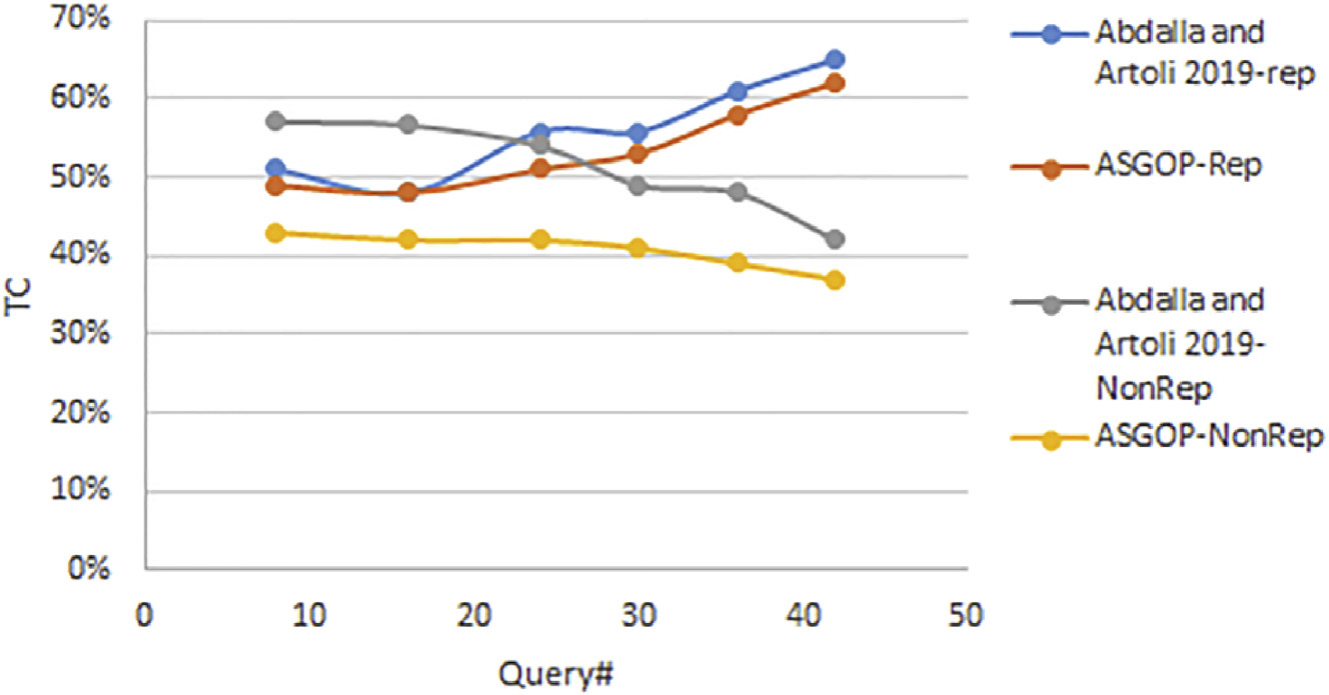
TC Percentage over all five Problems.

**Figure 12:**
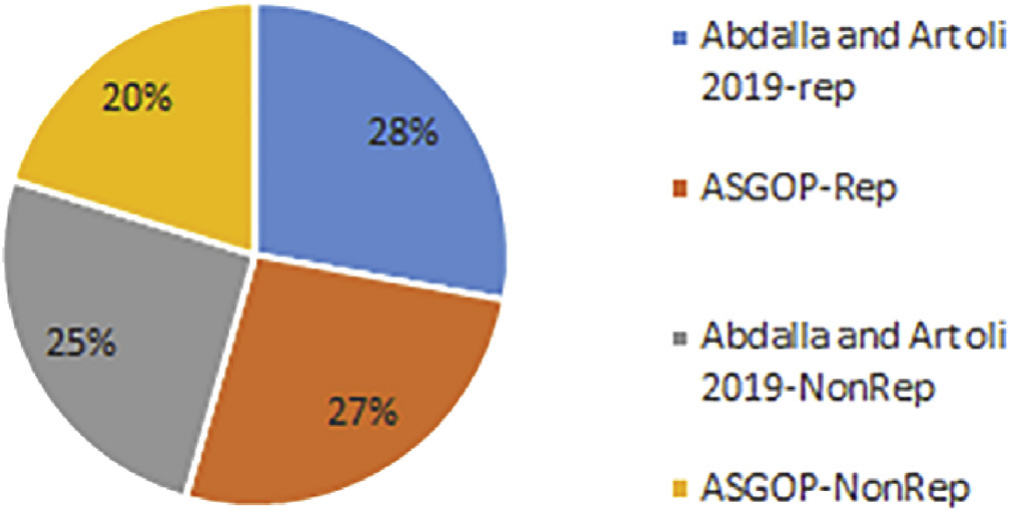
TC percentage in Average Over all five problems.

The final results are vividly illustrated in Figures 11 and 12. Figures showed that, over all problems, ASGOP outperforms (Abdalla and Artoli, 2019) slightly in terms of replication scenario. However, for the non-replication scenario, Figure 14 recorded that ASGOP outperforms (Abdalla and Artoli, 2019) maximally, and these findings are strongly backed by Figure 15 as all results were taken in averaged total.

In fact, the final results of experiments on the Employee dataset come to fully confirm the conclusions of results on the Ship dataset (Figures 6, 7, 8, 9, and 10). These results concluded that ASGOP significantly dominates both works in almost all aspects of DDBS design.

## 5 Discussion

As a matter of fact, in ASGOP (or its counterparts), three parameters have altogether been contributing to the overall performance of DDBS. Data fragmentation, as the first parameter, is performed either using one measure (hamming distance in (Sewisy et al., 2017; Abdalla and Artoli, 2019)) or as a combined measure “an aggregated similarity measure” in ASGOP. The site clustering, as the second parameter, is done differently in ASGOP and its peers, and finally, the data allocation, as the third parameter, which is already made using either a cost model or the greedy based algorithm. All of these parameters are examined severally in this section to examine the single impact of each parameter. In doing so, we can observe which parameter would have the greatest impact on DDBS performance. The undeniable impact of all parameters severally or combined is depicted briefly within the context of the Employee dataset (see section 4.5). Data fragmentation impact is implicitly drawn by the impact of similarity measures used to fragment data. This impact is explicitly represented by the number of clusters (data fragments) yielded by each work as given in Table 17. These statistics shows that ASGOP produces the minimum number of clusters (data fragments) comparing with (Abdalla and Artoli, 2019) with almost 26% reduction in the number of clusters. Despite the fact that, as mentioned earlier in section (4.5) (Sewisy et al., 2017), is being excluded as it was outperformed by (Abdalla and Artoli, 2019), we include it into Table 17 to just report that both works (Sewisy et al., 2017; Abdalla and Artoli, 2019) have the same number of clusters because they both used hamming distance to fragment data.

**Table 17:** Number of clusters (data fragments) produced by each work.

Approach/Problem#	P1	P2	P3	P4	P5	P6	P7	Average
Sewisy et al. (2017)	2	3	6	6	7	7	8	5.57
Abdalla and Artoli (2019)	2	3	6	6	7	7	8	5.57
ASGOP	2	3	4	4	5	5	6	4.14

**Figure 13:**
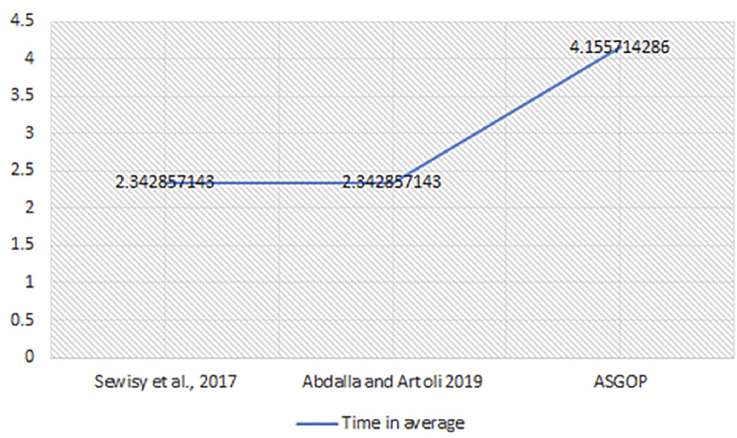
The average of time taken (over all problems) to find number of clusters in each work.

On the other extreme, the achievement of this parameter in ASGOP is in fact attributed to the effective use of the combined measure (hamming + nearby) instead of using a standalone measure (hamming). The combined measure contributes remarkably in finding the highly-tightened clusters (data fragments) which in turn led to ASGOP's outperformance. Nevertheless, the time taken (in seconds) to find data clusters was seen less in (Sewisy et al., 2017; Abdalla and Artoli, 2019) as they both used one measure to fragment data while ASGOP used combined measure. The combined measure hence needs more time to find the combination, and then fragment data in consequence (see Figure 13). Albeit the fact that ASGOP saved a computation time by applying the clustering algorithm directly on query set and eliminating the process of finding the numerical pattern of queries. Still, using double measures makes both works (Sewisy et al., 2017; Abdalla and Artoli, 2019) significantly faster than ASGOP. This tradeoff is therefore seen unescapable between both works. In the sense that in ASGOP, we are looking to reduce TC so we chose fragments number minimization over the speed needed to find these fragments. The price is then reserved by the smaller number of highly-similar fragments produced by ASGOP which makes sense of using combined measure. In consequent, producing the minimum number of data fragments meant a less complexity and traffic are needed for fragments to be scattered over sites/clusters. This would then lead to gaining the minimum number of visits (less traffic over the network) taken by each distributed query over site(s)/cluster(s) just to be answered. That is one convincing reason for ASGOP supremacy.

**Figure 14:**
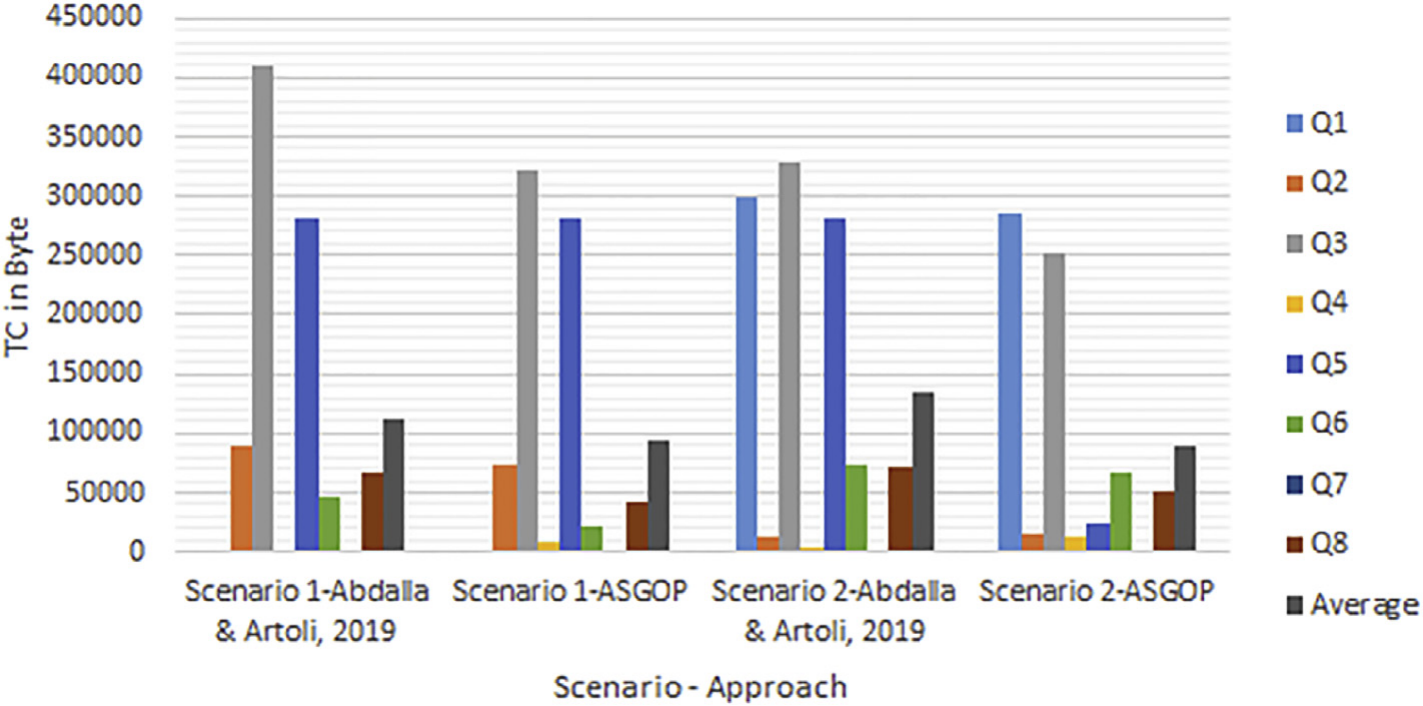
Problem 1, Transmission costs without Site Clustering.

**Figure 15:**
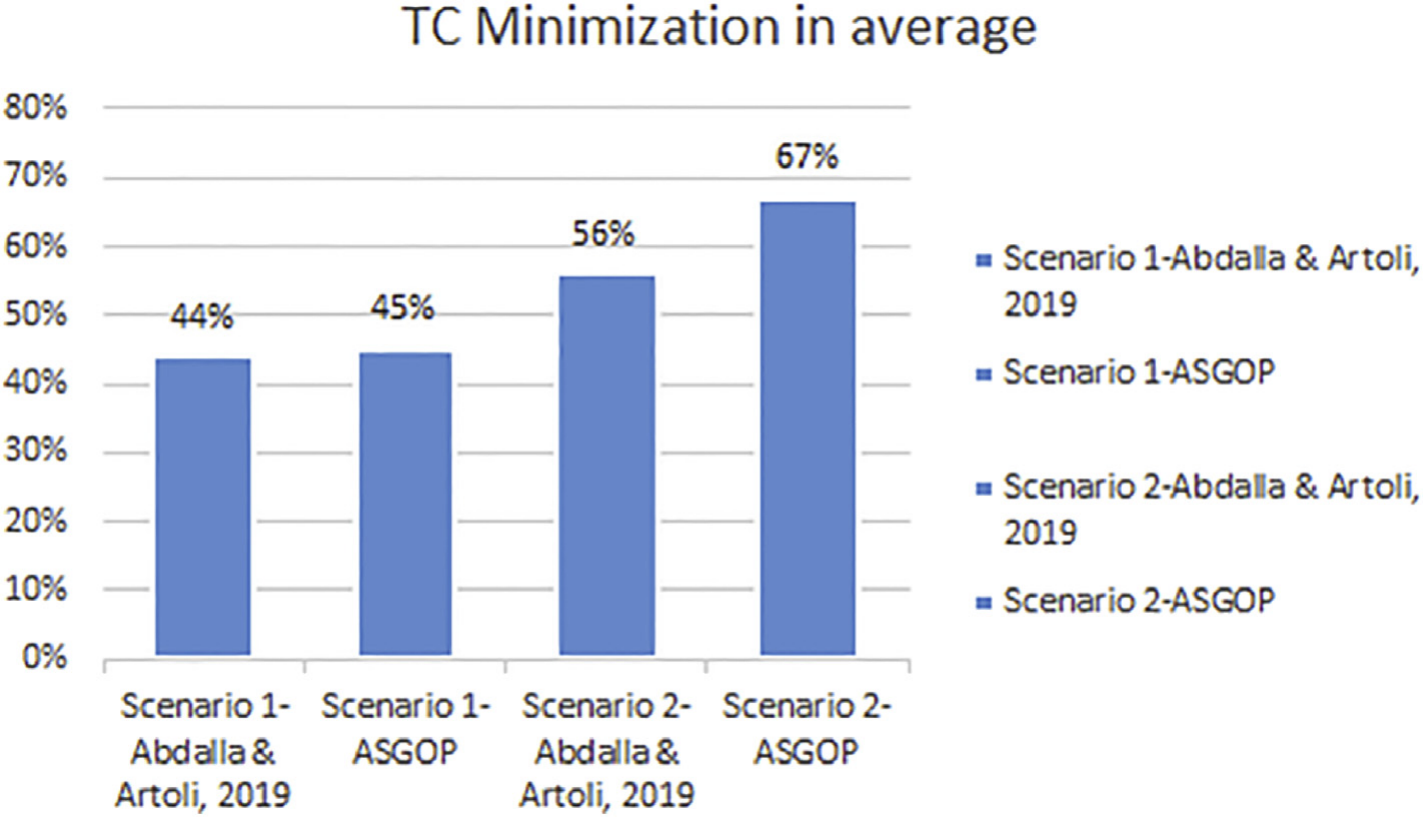
TC average for all problems, Transmission costs without Site Clustering.

**Figure 16:**
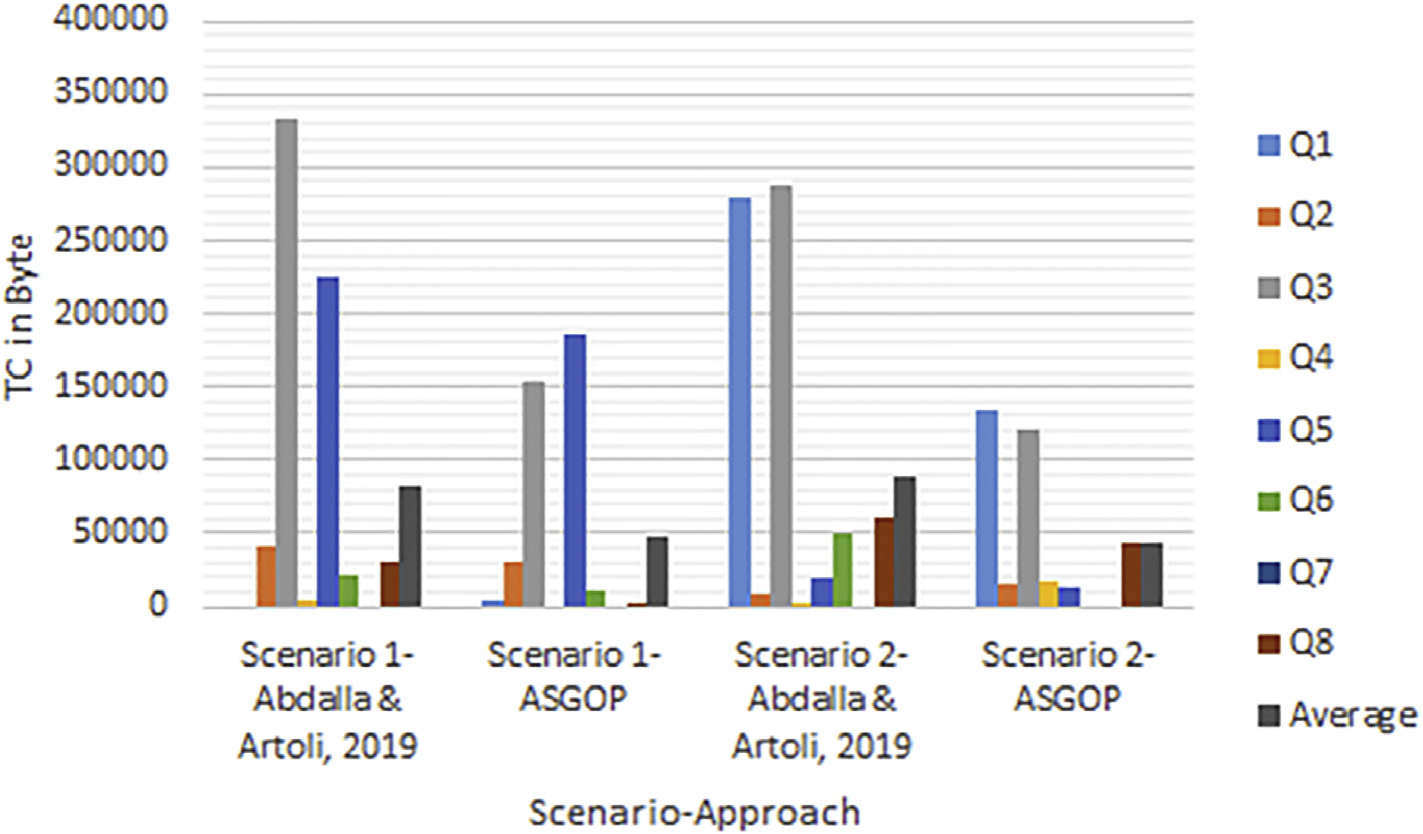
Problem 1, Transmission costs with Site Clustering.

**Figure 17:**
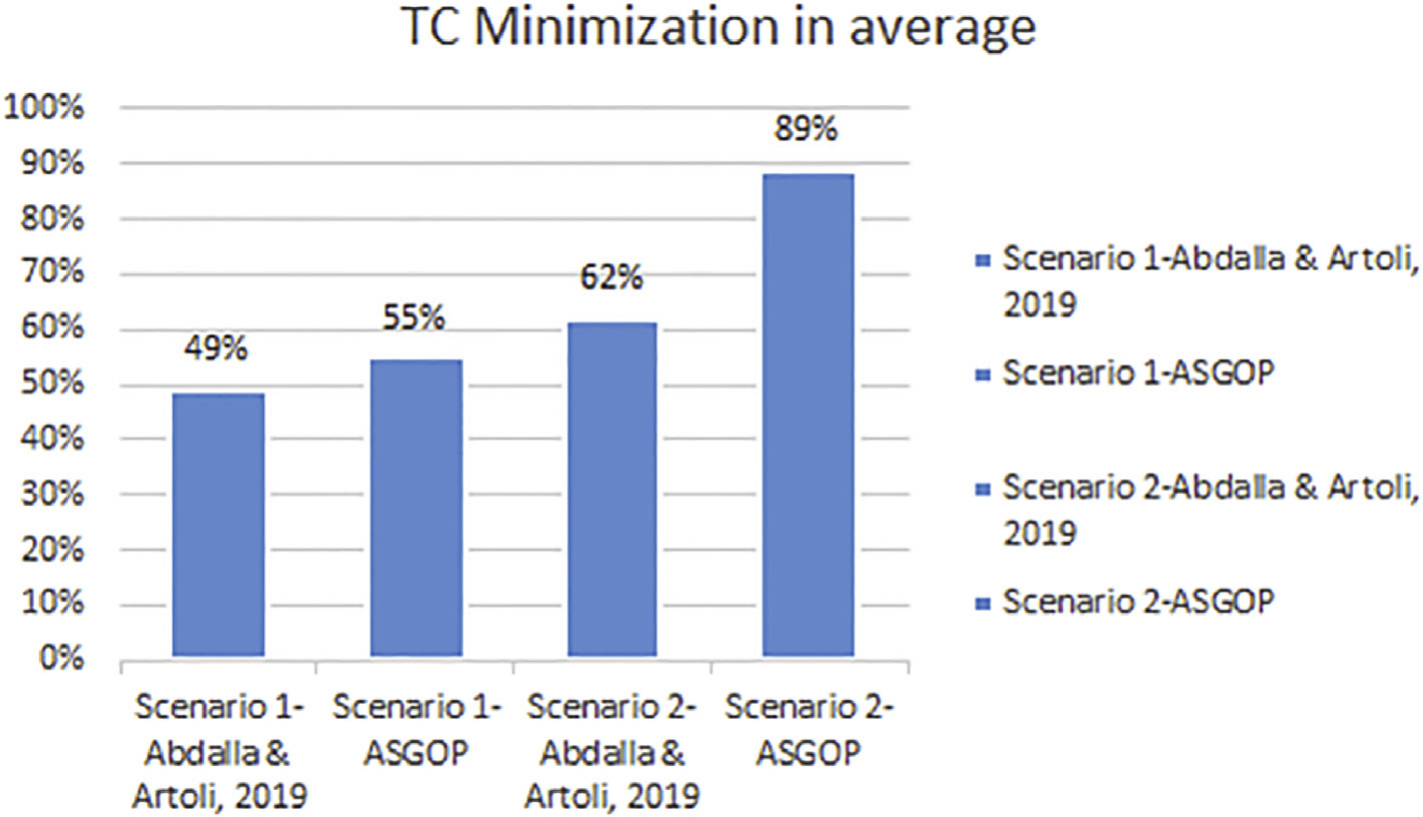
Average for all problems, Transmission costs with Site Clustering.

On the other hand, the second parameter which is the site clustering is implemented in (Abdalla and Artoli, 2019) and ASGOP only. We already excluded (Sewisy et al., 2017) from the further process due to its being inferior to (Abdalla and Artoli, 2019). The results showed that the clustering-based approach outperformed the non-clustering based approach for both works (see Figures 14, 15, 16, and 17). To confirm these claims, this parameter is also examined on both works to oversee the performance with and without site clustering as given in Figures 14, 15, 16, and 17.

From Figure 14, it is clear that even when sites were not clustered, ASGOP outperforms (Abdalla and Artoli, 2019) slightly in both scenarios. ASGOP behaves better in Q2, Q3, Q6, and Q8 and in the average of all queries as well. Q1 and Q7 were assigned zero values in both works as it was executed locally which means no TC incurred. On the other hand, Q4 and Q5 were in favor of (Abdalla and Artoli, 2019). By following the same pattern of the experiment (1), we took the average of all experiments to surprisingly note that ASGOP still behaves better in both scenarios with 45% and 67% over (Abdalla and Artoli, 2019) which recorded 44% and 56% for scenario (1) and scenario (2) respectively. For scenario (1), both works were so close from each other with a “1%” increase in TC minimization for ASGOP, though.

Like Figure 14, Figure 15 comes to confirm the fact that ASGOP still has the lead over (Abdalla and Artoli, 2019) when sites are being clustered. ASGOP behaves much better in Q_2_, Q_3_, Q_4_, Q_5_, Q_6_, Q_8,_ and in the average of all queries as well. For scenario 1, Q_1_ and Q_7_ were assigned zero value in (Abdalla and Artoli, 2019) as it was executed locally which means no TC incurred. On the other hand, Q_4_ and Q_5_ were in favor of (Abdalla and Artoli, 2019). For scenario (2), except Q_4_, ASGOP was super effective in reducing TC. By following the same pattern of experiment (1), we took average of all experiments to amazingly see that ASGOP still behaves much better in both scenarios with 55% and 89% over (Abdalla and Artoli, 2019) which recorded 49% and 62% for scenario (1) and scenario (2) respectively.

Furthermore, it is observed that the clustering based ASGOP outweighs the non-clustering based ASGOP by 42% in the average over all problems. This fact comes in complete agreement with results drawn in (Sewisy et al., 2017). It is also noted that site clustering has the greatest impact on DDBS performance as it contributes remarkably in promoting the overall DDBS performance. Figures 14 and 15 show that both works behave closely to each other. However, Figures 16 and 17 show that when the sites are being clustered, the difference in the performance of both works has been clearly marked with ASGOP being superior. This, in fact, could be attributed in one way or another to the use of both the aggregated similarity measure and the greedy base algorithm adopted in ASGOP to solve the DAP problem compared to the cost model used in (Abdalla and Artoli, 2019). As a matter of fact, as expected in terms of site clustering impact, these results agree entirely with results drawn in (Sewisy et al., 2017) which previously determined the greatest impact of site clustering on DDBS performance.

The third parameter is the data allocation which is already drawn in terms of the whole "building block" design of DDBS. We performed the whole design one time using the cost model of (Abdalla and Artoli, 2019) and another time using the greedy based approach (ASGOP). According to results, which are already drawn above in Figures 6, 7, 8, 9, 10, 11, and 12; 16–17, ASGOP outperforms (Abdalla and Artoli, 2019) in all aspects. In some cases, however, both approaches showed to be close to each other specifically for the replication scenario. Hence, using the greedy based approach has been an obvious reason for ASGOP supremacy that led to ASGOP yielding minimum TC which is essentially the objective function of all works.

**Lastly but not least**, based on the above concisely-drawn results and discussion, it can be confidently concluded that the data replication has a demoralizing impact on TC minimization, chiefly as update queries occupy the larger space of considered queries. The drawn results in Figures 6, 8, and 10 revealed that the replication scenario is the best choice when retrieval queries rate is larger than the update rate as it was the case in (P_1_–P_3_). However, this scenario started to negatively affect TC when update queries significantly grew as given in problems (P_4_–P_7_). On the other hand, the non-replication scenario was recorded to be by far the best option specifically when update queries made the large percentage of considered queries (P_4_–P_7_). This fact is reinforced when several problems were addressed for all considered works as shown in Figures 7, 9, and 11. Furthermore, these findings come to confirm the validity of the results of (Sewisy et al., 2017; Abdalla and Artoli, 2019) in terms of both replication and non-replication scenarios. In addition to that, it is worth stressing that the results of ASGOP come in full consistency with (Amer, 2018) with respect to the impact of data replication on DDBS performance.

To sum up, from the drawn-above discussion, three points could be deduced as follows;

- For the replication-based scenario, whenever sites, their relative clusters and considered queries are steadily growing with update queries being the larger space of all considered queries, TC is progressively maximized along with substantial degradation recorded for DDBS performance. These findings are supported by the drawn results of experiments conducted for the replication-based scenarios of both works ASGOP and (Sewisy et al., 2017).
- For the non-replication-based scenario, whenever sites, their relative clusters and considered queries are steadily swelling, whether update queries occupy a larger space of all considered queries or not, TC is imperceptibly maximized along with slight degradation observed at DDBS performance. These findings are supported by the drawn results of experiments conducted for both non-replication based scenarios of both ASGOP and (Abdalla and Artoli, 2019). Moreover, the non-replicated data allocation scenario of ASGOP has been proven to be the most effective scenario in all circumstances due to the greedy-nature algorithm of ASGOP. Consequently, this scenario can be selected to be incorporated in DDBS design specifically as update queries are significantly increased.
- Theoretically and empirically, compared to (Sewisy et al., 2017; Abdalla and Artoli, 2019), ASGOP used an aggregated similarity measure to: (1) reinforce the latent similarity between queries, (2) eliminate the need to QACM-producing process which led to reduce the computation time. Consequently, AHC was applied directly on the distance matrix which obtained using the proposed aggregated similarity measure. Instead of direct use of cost model to solve the data allocation, ASGOP pursued to solve the model itself using dynamic programming and it is proven practically effective. Lastly, according to the evaluation section, ASGOP has been successful at securing significantly better results than its peers. Most importantly, while both (Sewisy et al., 2017; Abdalla and Artoli, 2019) used **two** cost models to perform fragmentation and allocation, ASGOP leveraged **one** cost model to solve the data allocation using a greedy algorithm.

## 6 Conclusions and future work

This work comes with the main task embedded at presenting a well-articulated solution for DDBS design. It was primarily proposed with the key aim of highly reducing communication cost among network sites. An aggregated similarity-based hierarchical clustering algorithm for queries was developed to fragment data. The presented fragmentation procedure was set to be used in the context of the relational database, at the initial and later stages of DDBS design. The aggregated similarity struggled to find and reinforce the exact match between the considered queries so as to each cluster would contain only those highly-related queries. Then, to allocate fragmented data, a greedy-driven data allocation process was evolved. For the site clustering process, it was accomplished in the manner that ensures using the hierarchical clustering along with utilizing the concept of LDV proposed in (Amer et al., 2017).

On the other hand, for data allocation procedure, each fragment was decisively allocated into the site of minimum TC at each cluster on the basis of the Knapsack-inspired algorithm of greedy nature. In the sense that fragment would not be given to the targeted site unless it is guaranteed that no competitive site of minimum TC was found. That is, whenever the site of the lowest value of TC was identified; it was set to be the only container for that fragment within clusters individually. Two scenarios were considered for data allocation. In the first scenario, each fragment was allocated redundantly to all clusters and then to its best-fitting site in each relative cluster providing that objective function (TC) has been minimized. In the second scenario, in contrast, each fragment was assigned to one site (of the lowest TC value) among all sites of the network. Through this paper, several practical and empirical experiments have been conducted for the present work of this paper (ASGOP) and both (Sewisy et al., 2017; Abdalla and Artoli, 2019). The results were evaluated against each other in the purpose of verifying the mechanism of all works on the two data allocation scenarios under several circumstances. All the experimental results came in favor of ASGOP, specifically when the non-replication based scenario was adopted. The creativity of ASGOP lied in the proposed procedure of greedy-natured data allocation as each data fragment initially assigned to each site, the most-frequently-used queries then exposed on that site and concerned fragment at the same time. Consequently, among all sites of the network, that fragment was permanently given to the site of the lowest transmission costs (TC). According to the evaluation section, this step contributed remarkably at both decreasing TC and increasing DDBS performance as distributed queries processed were processed.

Finally, all parameters (data fragmentation, data allocation, and site clustering) that contribute to building the whole design of DDBSs has been severally examined. The examination is dedicated to identifying the parameter of the greatest impact on DDBS performance. Surprisingly, according to the drawn-above concisely-made discussion, site clustering has been the parameter of greatest impact with reduction reach almost 89% in TC. Experiments have been conducted with and without using site clustering to assert this claim which is proven completely correct. The next parameter has been the whole building block (all parameters) and clearly reflected on the overall performance of DDBS. Data fragmentation has occupied the third order in terms of impact on DDBS performance with a 26% reduction in TC.

### 6.1 Future work

While doing this research paper, an important limitation has been noted. This limitation represented in the fact that neither ASGOP nor did (Sewisy et al., 2017; Abdalla and Artoli, 2019) study the behavior of join-based queries. That is due to the fact that this type of query is of high-cost operations. In the follow-up work, in consequence, it is set to investigate the impact of such queries on DDBS performance. Moreover, the design of DDBS using K-means is going to be investigated and compared with the hierarchical clustering-based design.

## Declarations

### Author contribution statement

Ali A. Amer: Conceived and designed the experiments; Performed the experiments; Analyzed and interpreted the data; Contributed reagents, materials, analysis tools or data; Wrote the paper.

Marghny H. Mohamed: Performed the experiments; Contributed reagents, materials, analysis tools or data; Wrote the paper.

Khaled Abdullah Al_Asri: Conceived idea; Analyzed and interpreted the data; Contributed reagents, materials, analysis tools or data.

### Funding statement

This research did not receive any specific grant from funding agencies in the public, commercial, or not-for-profit sectors.

### Competing interest statement

The authors declare no conflict of interest.

### Additional information

No additional information is available for this paper.
